# Smart Biosensing Nanomaterials for Alzheimer’s Disease: Advances in Design and Drug Delivery Strategies to Overcome the Blood–Brain Barrier

**DOI:** 10.3390/bios16010066

**Published:** 2026-01-21

**Authors:** Manickam Rajkumar, Furong Tian, Bilal Javed, Bhupendra G. Prajapati, Paramasivam Deepak, Koyeli Girigoswami, Natchimuthu Karmegam

**Affiliations:** 1 Department of Biotechnology, Karpagam Academy of Higher Education, Coimbatore 641021, Tamil Nadu, India; 2 Center for Cancer Research, Karpagam Academy of Higher Education, Coimbatore 641021, Tamil Nadu, India; 3 School of Food Science and Environmental Health, Technological University Dublin, Grangegorman, D07 ADY7 Dublin, Ireland; 4 Nanolab Research Centre, Physical to Life Sciences Research Hub, Technological University Dublin, Camden Row, D08 CKP1 Dublin, Ireland; 5 Department of Pharmaceutics, Parul Institute of Pharmacy, Faculty of Pharmacy, Parul University, Waghodia, Vadodara 391760, Gujarat, India; 6 Department of Life Sciences, School of Biological and Forensic Sciences, Kristu Jayanti University, Bengaluru 560077, Karnataka, India; 7 Center for Global Health Research, Saveetha Medical College and Hospital, Saveetha Institute of Medical and Technical Sciences (SIMATS), Saveetha University, Chennai 602105, Tamil Nadu, India; 8 PG and Research Department of Botany, Government Arts College (Autonomous), Salem 636007, Tamil Nadu, India

**Keywords:** nanomaterials, Alzheimer’s disease, drug delivery system, blood–brain barrier

## Abstract

Alzheimer’s disease (AD) is a progressive neurodegenerative disorder marked by persistent memory impairment and complex molecular and cellular pathological changes in the brain. Current treatments, including acetylcholinesterase inhibitors and memantine, only help with symptoms for a short time and do not stop the disease from getting worse. This is mainly because these drugs do not reach the brain well and are quickly removed from the body. The blood–brain barrier (BBB) restricts the entry of most drugs into the central nervous system; therefore, new methods of drug delivery are needed. Nanotechnology-based drug delivery systems (NTDDS) are widely studied as a potential approach to address existing therapeutic limitations. Smart biosensing nanoparticles composed of polymers, lipids, and metals can be engineered to enhance drug stability, improve drug availability, and target specific brain regions. These smart nanoparticles can cross the BBB via receptor-mediated transcytosis and other transport routes, making them a promising option for treating AD. Additionally, multifunctional nanocarriers enable controlled drug release and offer theranostic capabilities, supporting real-time tracking of AD treatment responses to facilitate more precise and personalized interventions. Despite these advantages, challenges related to long-term safety, manufacturing scalability, and regulatory approval remain. This review discusses current AD therapies, drug-delivery strategies, recent advances in nanoparticle platforms, and prospects for translating nanomedicine into effective, disease-modifying treatments for AD.

## 1. Introduction

Alzheimer’s disease (AD) is a prominent neurodegenerative disorder marked by progressive deterioration of memory, cognitive abilities, and related behavioral and psychological symptoms of dementia [1]. The prevalence of AD rises substantially with advancing age, predominantly impacting individuals over 60 years. With the rapid aging of the global population, the number of individuals affected by AD is projected to reach approximately 131 million by 2050. The World Health Organization (WHO) predicts that AD will likely become the fourth leading cause of death by 2030 due to the lack of effective diagnostic and treatment options [2]. The substantial personal, family, and socioeconomic challenges associated with AD highlight the urgent need for advances in managing the disease. Although significant progress has been made in understanding the mechanisms behind AD, its exact pathogenesis remains unclear, posing a substantial obstacle to developing effective therapies. Numerous hypotheses have been proposed to explain the disease’s development, each emphasizing different pathological hallmarks of onset and progression that could serve as potential therapeutic targets [3,4].

Early detection of AD and prompt interventions are crucial yet unresolved challenges in its management. The ideal therapeutic window—especially during the preclinical symptomatic phases—remains unclear due to the lack of reliable confirmatory diagnostic tools [5]. Current diagnostic methods mainly focus on identifying pathological biomarkers, such as amyloid-β (Aβ) aggregates and hyperphosphorylated tau (p-tau), which form amyloid plaques and neurofibrillary tangles (NFTs), respectively, the hallmark features of AD. However, assessments of cerebrospinal fluid (CSF) and neuroimaging techniques provide only supportive, rather than definitive, diagnostic information, as the relationship between biomarker levels and disease progression remains debated. Therefore, integrating multimodal diagnostic approaches that combine biochemical, imaging, and genetic markers could significantly enhance diagnostic accuracy and facilitate early therapeutic interventions [6,7]. In this context, theranostic methods that integrate therapeutic and diagnostic functions into a single platform hold considerable promise by enabling real-time monitoring of drug efficacy and disease progression. Despite some encouraging early results from a limited number of theranostics agents, this field remains relatively new and requires extensive research before widespread clinical use. Additionally, the Blood–Brain Barrier (BBB), a highly selective and dynamic neurovascular interface comprising endothelial cells, pericytes, astrocytes, and supporting glial cells, presents a significant obstacle for effective AD treatment by blocking most therapeutic agents from reaching the brain [8,9].

**Figure 1:**
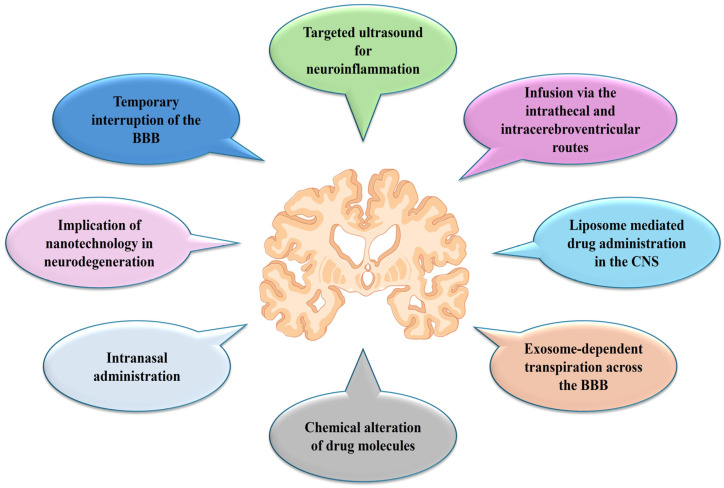
Overview of AD pathology and prominent methods employed to transport therapeutics through the BBB. The illustration emphasizes significant obstacles and innovative nanotechnology-based strategies for successful brain targeting. (Created in BioRender, 2025).

The build-up of extracellular Aβ plaques and NFTs made of hyperphosphorylated tau is a hallmark of AD pathogenesis. These aggregates impede synaptic transmission and disturb the integrity of neuronal microtubules. Neuronal loss and cognitive decline are also caused by oxidative stress, mitochondrial dysfunction, and neuroinflammation [10]. Despite significant advances in elucidating genetic risk factors and molecular pathways, the precise etiology of AD remains unresolved. Current treatments primarily provide symptomatic relief without modifying disease progression. Recently developed disease-modifying antibodies, such as aducanumab, lecanemab, and donanemab, which target Aβ aggregates, have demonstrated potential to slow cognitive decline; however, their long-term safety and efficacy remain under evaluation. The BBB presents a significant obstacle, blocking over 98% of small molecules and nearly all macromolecular therapeutics, thereby underscoring the urgent need for innovative drug delivery strategies [11,12]. Only a small proportion of hydrophobic molecules can cross the BBB, limiting the delivery of many potentially effective therapeutics. Nanoparticle-based drug delivery systems represent a promising approach to enhance drug stability, increase BBB permeability, and facilitate targeted administration to affected neuronal regions. The application of nanotechnology in future AD therapies may facilitate not only symptomatic relief but also direct intervention in underlying molecular mechanisms to modify disease progression [13]. Biosensor technologies have recently emerged as effective tools for early diagnosis and monitoring of AD by enabling sensitive detection of key biomarkers, including Aβ peptides, tau protein, and phosphorylated tau. Smart nanomaterials are central to advancing these biosensors, as they enhance biorecognition efficiency, signal transduction, and analytical sensitivity through their high surface area, distinctive electrical properties, and biocompatibility [14]. In addition to improving biosensor performance, smart nanomaterials facilitate the development of multifunctional theranostic platforms that combine biomarker detection with targeted drug delivery across the BBB. Consequently, progress in nanomaterial design is intrinsically linked to biosensor development, underscoring the importance of nanotechnology-driven biosensing strategies for AD diagnosis and therapeutic intervention [15]. Figure 1 presents emerging strategies to enhance drug delivery across the BBB in AD.

These strategies include transient BBB disruption and focused ultrasound to enable controlled, localized therapeutic delivery to the brain. Alternative administration routes, such as intranasal delivery, offer a noninvasive pathway that bypasses systemic circulation and directly targets the central nervous system. Chemical modification of drug molecules increases BBB permeability, stability, and pharmacokinetics. Nanotechnology-based platforms, including liposomes, polymeric nanoparticles, and exosome-mediated transport systems, support targeted and sustained drug delivery to affected brain regions. Additionally, direct administration methods, such as intrathecal and intracerebroventricular infusion, provide precise delivery to the cerebrospinal fluid. These approaches address BBB-associated limitations and seek to improve the efficacy of targeted therapeutic interventions for AD [8,16].

Smart nanomaterial-based delivery systems offer a flexible and promising approach to overcoming the BBB’s barriers in AD treatment. Nanoparticle-based platforms have the potential to significantly improve therapeutic efficacy and clinical outcomes by enhancing drug stability, improving targeting specificity, and enhancing brain penetration [17]. This review summarizes recent advances in nanoparticle-mediated strategies, with particular emphasis on their role in elucidating AD pathogenesis through interactions with the brain microenvironment. Nanocarriers such as liposomes, polymeric nanoparticles, and inorganic nanomaterials increase drug bioavailability and stability and enable controlled release within specific brain regions. These systems interact with Aβ aggregates, reduce oxidative stress, and modulate neuroinflammatory pathways. Additionally, multifunctional nanomaterials facilitate integrated diagnostic and therapeutic applications. Rigorous assessment of biocompatibility, toxicity, and translational feasibility highlights the potential of nanomedicine to advance safe and effective AD treatment strategies [18].

## 2. Risk Factors, Challenges, and Hypothesis of AD

AD is caused by a complex interplay of lifestyle, environmental, and genetic factors. The greatest risk factor is age, with a marked increase in incidence among individuals aged 65 and older. Genetic predisposition, particularly mutations in APP, PSEN1, PSEN2, and the presence of the APOE ε4 allele, increases susceptibility [19]. Additional risk factors include traumatic brain injury, cardiovascular and metabolic disorders such as hypertension and diabetes, chronic infections, and prolonged exposure to environmental toxins, including heavy metals. These factors contribute to the neuropathological hallmarks of AD: extracellular Aβ plaque deposition and intracellular neurofibrillary tangle (NFT) accumulation composed of hyperphosphorylated tau protein [20,21]. The aggregation of these proteins initiates a cascade of pathological events, including mitochondrial dysfunction, oxidative stress, neuroinflammation, synaptic loss, and progressive neuronal death, ultimately leading to widespread cortical atrophy and brain shrinkage. Mechanistic hypotheses include the cholinergic hypothesis, which focuses on impaired acetylcholine neurotransmission, and the amyloid cascade hypothesis, which emphasizes aberrant Aβ production and aggregation as early pathogenic events. Nevertheless, no single hypothesis fully accounts for AD pathogenesis, underscoring the complex interplay of molecular pathways, environmental exposures, and genetic susceptibilities in disease onset and progression [22,23].

### 2.1. Amyloid Cascade Hypothesis

**Figure 2:**
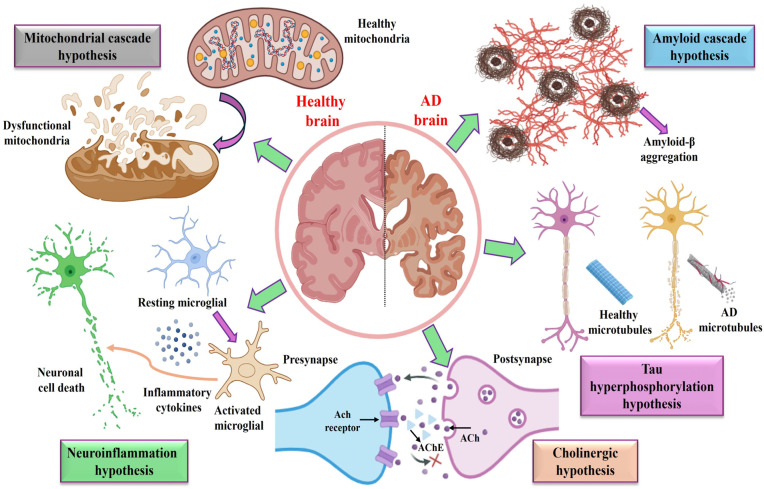
This schematic illustrates key pathological theories of AD, including mitochondrial dysfunction, Aβ accumulation, neuroinflammation, tau hyperphosphorylation, and cholinergic deficits. The comparison between a healthy brain and one affected by AD underscores neuronal deterioration, synaptic loss, and microtubule instability. The interplay among dysfunctional mitochondria, activated microglia, and Aβ accumulation drives both inflammatory and neurodegenerative processes. Collectively, these mechanisms illustrate the complex progression of AD. (Created in BioRender, 2025).

Introduced in the early 1990s, the amyloid cascade hypothesis remains one of the most influential and widely accepted models for explaining the underlying pathogenesis of AD. It proposes that a disruption of the balance between Aβ production and clearance leads to accumulation and aggregation of Aβ in the brain, triggering a cascade of neurodegenerative processes, including oxidative stress, inflammation, synaptic dysfunction, and neuronal death [24,25]. Aβ peptides, mainly Aβ_40_ and Aβ_42_, are produced from the amyloid precursor protein (APP) through a two-step cleavage involving β- and γ-secretases. While Aβ_40_ accounts for the majority of Aβ species, Aβ_42_ is more prone to aggregation and exhibits greater neurotoxicity, thereby playing a crucial role in plaque formation. Consistent with this hypothesis, many therapeutic strategies have aimed to reduce Aβ production, prevent aggregation, or enhance plaque clearance. However, even after removing Aβ plaques in clinical trials, most Aβ-targeting drugs have not shown significant cognitive improvements, raising questions about the validity of this hypothesis [26,27]. Emerging perspectives suggest that soluble Aβ oligomers, rather than mature fibrils or plaques, are the primary neurotoxic agents linked to synaptic dysfunction and cognitive decline (Figure 2). Consequently, early-stage interventions targeting Aβ oligomers may prove more promising therapeutic options. These oligomers could also serve as more reliable biomarkers for diagnosing AD and monitoring its progression [28].

### 2.2. Tau Hypothesis

The tau hypothesis proposes that hyperphosphorylation of the tau protein is a key factor in the development of AD. Tau, a protein linked to microtubules, is crucial for microtubule formation and maintaining the stability of the neuronal cytoskeleton. Its activity is carefully regulated by reversible phosphorylation; however, in disease states, the balance between phosphorylation and dephosphorylation is disrupted, leading to excessive tau phosphorylation [29]. When tau becomes hyperphosphorylated, it detaches from microtubules and forms aggregates known as paired helical filaments and NFTs, which impair microtubule dynamics, disrupt axonal transport, and ultimately cause neuronal dysfunction and cell death [30] (Figure 2). Research shows that levels of p-tau and NFTs are more strongly linked to cognitive decline and memory issues than amyloid plaques, indicating that tau pathology plays a central role in disease progression. Consequently, therapies targeting tau—such as inhibiting tau phosphorylation, preventing tau aggregation, and promoting tau clearance—are currently under investigation, with several candidates in clinical trials [31]. Additionally, measurements of tau and p-tau in cerebrospinal fluid and plasma serve as valuable biomarkers for diagnosing AD and tracking disease progression. Nonetheless, the lack of specific tau imaging probes limits the ability to assess tau-focused treatments. Ongoing research into tau pathology could enhance diagnostic accuracy and treatment effectiveness in managing AD [32].

### 2.3. Cholinergic Hypothesis

According to the cholinergic hypothesis, the cognitive deficits observed in AD are largely attributable to the death of cholinergic neurons and reduced acetylcholine (ACh) levels [33]. Reduced activity of key cholinergic markers, such as choline acetyltransferase (ChAT) and acetylcholinesterase (AChE), has been linked to disrupted synaptic transmission and memory problems. Because ACh is essential for learning and thinking, various drugs have been developed to enhance cholinergic signaling by inhibiting AChE, adding cholinergic precursors, or activating cholinergic receptors [34] (Figure 2). Four cholinesterase inhibitors—donepezil, rivastigmine, galantamine, and tacrine—have been approved by the FDA. However, these drugs only provide temporary relief of symptoms without altering disease progression, suggesting that cholinergic dysfunction may be a secondary factor rather than the leading cause of AD. The metal ion hypothesis supports this idea by proposing that imbalances in metal ions, particularly copper, zinc, and iron, contribute to the development of AD [35,36]. Higher levels of metal ions in amyloid plaques promote Aβ aggregation, oxidative stress, and neuron damage. As a result, metal chelation therapy has been explored to restore metal balance and reduce neurotoxicity. Still, achieving treatment selectivity without disrupting essential metal-dependent functions remains a key challenge for clinical use [37].

### 2.4. Oxidative Stress, Neuroinflammatory, and Mitochondrial Cascade Hypotheses

Oxidative stress (OS) is a central feature of AD pathology, arising from an imbalance between excessive reactive oxygen species (ROS) and inadequate antioxidant defenses. The OS hypothesis asserts that oxidative stress is instrumental in AD pathogenesis by facilitating Aβ accumulation, tau hyperphosphorylation, mitochondrial dysfunction, and abnormal metal ion metabolism. Impaired mitochondrial electron transport, in conjunction with redox-active metals, elevates ROS production and establishes a self-perpetuating cycle of oxidative damage that accelerates neurodegeneration [38]. Neuroinflammation, which is closely associated with oxidative stress, also contributes significantly to AD. Activated astrocytes and microglia secrete pro-inflammatory cytokines, including tumor necrosis factor-α (TNF-α), interleukin (IL)-1β, and IL-18, in response to Aβ deposits and phosphorylated tau, thereby exacerbating neuronal injury through sustained inflammatory signaling. Consequently, anti-inflammatory therapies have been investigated as potential interventions to disrupt this deleterious cycle. The mitochondrial cascade hypothesis proposes that both genetic and environmental factors modulate mitochondrial resilience and its progressive decline with age [39,40]. Once mitochondrial dysfunction exceeds a critical threshold, hallmark AD symptoms such as Aβ accumulation, tau tangles, and synaptic loss manifest. In contrast, the secondary mitochondrial cascade model posits that mitochondrial dysfunction is a downstream consequence of Aβ toxicity, underscoring the multifaceted nature of AD progression [41,42] (Figure 2).

## 3. Blood–Brain Barrier

**Figure 3:**
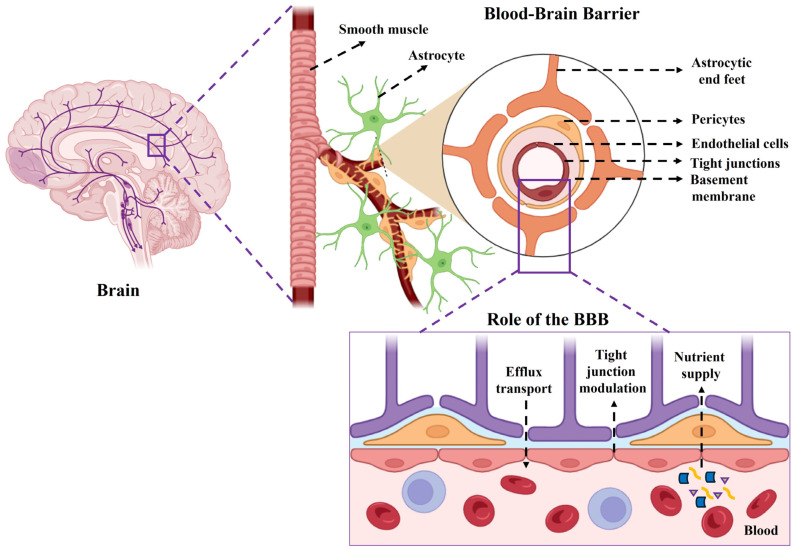
This figure represents the structural composition of the BBB, highlighting endothelial cells connected by tight junctions and supported by pericytes and astrocytic end-feet. The BBB functions as a selective barrier that regulates the transport of substances between the blood and the brain. It ensures nutrient supply, maintains ionic balance, and utilizes efflux transporters to eliminate harmful compounds. Through these coordinated activities, the BBB maintains neural homeostasis and safeguards the CNS from toxins, pathogens, and fluctuations in circulating bioactive molecules. (Created in BioRender, 2025).

The BBB is a dynamic, highly selective physiological interface made up of endothelial cells connected by tight junctions that prevent drugs from passively diffusing into the brain from the bloodstream. Although the BBB is essential for maintaining CNS homeostasis, it presents a significant obstacle to therapeutic delivery in neurological diseases. Dysfunction of the BBB is marked by endothelial activation, disruption of tight junctions, and altered expression of transporters. These pathological alterations have prompted the development of both passive- and active-targeting nanocarrier-based delivery systems to enhance drug penetration into the brain while ensuring safety and translational feasibility [43]. Only small molecules with molecular weights below approximately 400 Da and fewer than eight hydrogen-bond donors and acceptors can cross the BBB via passive diffusion. While this selectivity preserves the neural microenvironment, it greatly restricts the delivery of hydrophilic drugs, peptides, and large imaging agents. Additionally, the BBB regulates ionic balance and nutrient transport, with endothelial ion channels and transporters precisely controlling the concentrations of Na^+^, K^+^, Ca^2+^, and Cl^−^ to maintain the electrochemical gradients necessary for neuronal signaling [37] (Figure 3).

Several specialized transport mechanisms regulate molecular movement across the BBB, including carrier-mediated transport (CMT), receptor-mediated transport (RMT), absorptive-mediated transport (AMT), and active efflux systems. Solute carrier (SLC) and ATP-binding cassette (ABC) transporters facilitate the transfer of nutrients, metabolites, and xenobiotics into and out of the brain [44]. For example, P-glycoprotein (P-gp) effluxes lipophilic drugs and toxins, whereas glucose transporter 1 (GLUT1) facilitates glucose uptake. RMT is based on specific ligand-receptor interactions, such as those involving transferrin receptors (TfR) and insulin receptors (IR), which are commonly utilized in drug delivery strategies. AMT, on the other hand, promotes caveolae-mediated endocytosis via electrostatic interactions between positively charged molecules and the endothelial glycocalyx [45,46]. Nanoparticles can utilize these transport pathways by coating their surfaces with ligands like glucose, mannose, amino acids, or cationic peptides, thereby enhancing recognition via GLUT, LAT, or CPP pathways and improving brain-targeted delivery for AD [47].

Systemic and direct CNS administration routes serve complementary functions in nanoparticle-based drug delivery for AD. Oral delivery of nanoparticle formulations enhances drug stability and intestinal absorption, but BBB penetration remains limited due to first-pass metabolism and restrictive endothelial barriers [48]. Intravenous administration, especially when combined with surface-engineered nanoparticles bearing targeting ligands, enables controlled systemic dosing and leverages receptor- or adsorptive-mediated transcytosis to improve BBB crossing and brain accumulation. Direct CNS routes, such as intrathecal and intracerebroventricular (ICV) administration, bypass the BBB and permit nanoparticles to access the cerebrospinal fluid, resulting in high local drug concentrations with reduced systemic exposure [49]. Despite their invasive nature, these routes are particularly advantageous for delivering biologics, gene therapies, and sustained-release nanocarriers, underscoring their significance for targeted and effective AD therapeutic interventions [50].

In addition to systemic delivery, various local and physical strategies have been developed to bypass the BBB. Direct methods, such as intrathecal and ICV methods, improve CNS access. While intracerebral injection allows for targeted, localized drug delivery, its diffusion remains limited to the area around the injection site [51]. Both the ICV and intrathecal routes enable broader distribution through cerebrospinal fluid, but are invasive and carry infection risks. Non-invasive techniques, particularly focused ultrasound (FUS) combined with microbubbles, provide a temporary, reversible method for opening the BBB. The movement of microbubbles in response to ultrasound generates mechanical stress, temporarily loosening endothelial tight junctions and allowing nanoparticles to safely penetrate brain tissue. Combining BBB-modulation techniques with advanced nanocarrier design offers a promising approach to enhance therapeutic precision, safety, and efficacy in the management of AD [52].

## 4. Current Treatments and Limitations of AD

Current therapeutic strategies for AD are primarily designed to alleviate symptoms rather than to halt or reverse the underlying neurodegenerative processes. Cholinesterase inhibitors, such as donepezil, rivastigmine, and galantamine, enhance cholinergic neurotransmission by inhibiting acetylcholinesterase, resulting in modest improvements in cognitive function and activities of daily living [53]. For example, rivastigmine has shown an average improvement of the ADAS-Cog scale compared with placebo, indicating a measurable but limited cognitive benefit [54]. Memantine, an NMDA receptor antagonist, reduces glutamate-mediated excitotoxicity and produces minor but meaningful enhancements in cognition and overall functioning. In addition to symptomatic management, recent research increasingly emphasizes disease-modifying therapies, including anti-amyloid and anti-tau strategies, immunotherapies, and neuroprotective agents, which aim to target the molecular mechanisms of AD and slow or prevent disease progression [55]. Monoclonal antibodies, such as aducanumab, have demonstrated dose-dependent reductions in amyloid-β plaque burden, along with improvements on the CDR-SB scale. Lecanemab has also been effective in reducing amyloid accumulation and delaying cognitive and functional decline in early-stage AD [56]. Therapies targeting tau pathology are emerging, with initial trials showing decreased tau levels and early cognitive improvements. Strategies for drug repurposing, such as using pioglitazone to modulate neuroinflammation and support synaptic function, hold additional promise. Additionally, new delivery systems, such as nanoparticle-based methods, may improve therapeutic transport across the BBB and enhance treatment effectiveness [57].

There are few and mainly symptomatic therapy options available for AD today. Although acetylcholinesterase inhibitors (AChEIs) enhance cholinergic neurotransmission, their use is often limited by gastrointestinal side effects and only modestly improves cognition [48,58]. Memantine is indicated for moderate to severe AD to mitigate glutamate-induced excitotoxicity; however, its clinical benefits are minimal, and it does not alter disease progression. There is growing interest in anti-amyloid monoclonal antibodies targeting Aβ plaques, a key feature of AD pathology [59]. Aducanumab, which received accelerated FDA approval in 2021, effectively reduces amyloid accumulation but shows variable cognitive outcomes and a high rate of amyloid-related imaging abnormalities, such as brain swelling and microhemorrhages [60]. High costs and the need for frequent MRI monitoring further challenge its therapeutic effectiveness. In 2023, lecanemab was approved, demonstrating a modest (~27%) slowing of cognitive decline and a significant reduction in amyloid burden in early-stage AD [61]. However, risks like ARIA and treatment expenses remain significant barriers. Despite extensive research into amyloid and tau pathology, clinical success is limited by poor drug penetration through the BBB. Efforts to improve delivery—via receptor-mediated transport, invasive methods, or temporary BBB disruption—have had limited success in clinical settings. These issues underscore the urgent need for innovative drug-delivery systems, such as nanotechnology-based approaches, to enhance therapeutic outcomes in AD [62].

One of the biggest challenges in developing effective treatments for AD is to address the BBB’s limiting nature while preserving its vital role in CNS homeostasis. These drawbacks underscore the need for novel therapeutic approaches that address the complexity of AD while ensuring efficacy and safety. Nanoparticle-based approaches have attracted considerable interest as promising solutions. Because of their small size, adjustable surface chemistry, and potential for targeted delivery, nanoparticles can penetrate the BBB more effectively than conventional therapies. These systems can deliver therapeutic agents directly to neuronal or molecular targets, influence disease pathways, and possibly reduce systemic side effects. By improving drug bioavailability and delivery precision, nanotechnology-based interventions offer a promising strategy to overcome current therapeutic challenges and advance the development of more effective treatments for AD [63].

## 5. Significance of Smart Nanoparticles

Smart nanomaterials are advanced nanoscale systems engineered to respond selectively to specific biological or physicochemical stimuli, thereby enabling controlled and targeted therapeutic delivery. Key properties, such as nanoscale dimensions, a high surface-to-volume ratio, tunable surface charge, and structural flexibility, collectively enhance interactions with biological environments. Stimuli-responsive behaviors, including sensitivity to pH, redox conditions, enzymatic activity, or external triggers, permit precise regulation of drug release at disease-specific sites [64]. In the context of AD, smart nanomaterials are developed to improve drug stability, increase brain bioavailability, and facilitate efficient transport across the BBB. Surface functionalization and adaptive responsiveness enable site-specific targeting, thereby reducing off-target effects and systemic toxicity. These materials also enable sustained, controlled release profiles, thereby maintaining therapeutic concentrations within the brain. Due to their multifunctional properties and adaptability, smart nanomaterials offer a promising strategy for developing effective, disease-modifying therapeutic approaches for AD [65,66].

Nanoparticles function as colloidal carriers that encapsulate therapeutic agents within a single, stable unit. This structure enables controlled release and targeted delivery in biological systems. In contrast, conventional drug formulations, including powders, tablets, capsules, and liquid suspensions, often exhibit limitations such as poor bioavailability, high dosing requirements, rapid first-pass metabolism, and suboptimal pharmacokinetic profiles [67]. In addition, numerous bioactive compounds, including polyphenols, proteins, and peptides, exhibit low solubility and limited gastrointestinal absorption, which diminishes therapeutic efficacy and may lead to clinical failure. NP-based delivery systems address these challenges by protecting encapsulated drugs from enzymatic degradation, enhancing membrane permeability, and facilitating sustained release, thereby enabling therapeutic effects at lower doses [68]. The tunable physicochemical properties of NPs, such as nanoscale dimensions (1–100 nm), high surface-to-volume ratios, and modifiable surface charges, enhance absorption, prolong circulation, and enable targeted transport across biological barriers, including the blood–brain barrier. These attributes position NPs as promising platforms for precision drug delivery and improved clinical outcomes [69]. In neurological applications, NPs offer a promising approach to bypass the BBB, a significant obstacle to CNS drug delivery. Surface modification strategies—such as increasing lipophilicity, reducing opsonization, or adding targeting ligands—aid in BBB penetration and improve brain bioavailability. Small molecules with molecular weights between 400 and 500 Da can cross the blood–brain barrier via passive diffusion. However, many therapeutic agents do not meet these criteria, which restricts their delivery to the central nervous system. Nanoparticle-based drug delivery systems, when appropriately designed, can address this limitation. These nanocarriers traverse the BBB via active mechanisms, including carrier-mediated transport, adsorptive-mediated transcytosis, and receptor-mediated endocytosis. Consequently, nanoparticle-based delivery represents a promising approach for precise, efficient, and sustained treatment of neurodegenerative diseases [70].

Smart nanomaterials utilized in AD therapy are broadly classified as polymeric, lipid-based, inorganic, and stimuli-responsive nanocarriers, each with distinct physicochemical and biological characteristics. Polymeric nanoparticles are extensively studied for their biodegradability, structural adaptability, and capacity to provide sustained, controlled drug release, thereby enhancing therapeutic stability. Lipid-based nanocarriers exhibit high biocompatibility and efficiently encapsulate hydrophobic drugs, thereby enhancing brain uptake and facilitating penetration of the BBB. Inorganic nanomaterials exhibit unique magnetic, optical, or catalytic properties that support both diagnostic and therapeutic applications [71,72]. However, potential issues related to long-term accumulation and toxicity require thorough assessment. Stimuli-responsive nanomaterials are designed to release therapeutic agents in response to specific biological signals, thereby enabling precise, site-specific drug delivery. Comparative analysis of these nanomaterial categories is critical for elucidating their respective benefits and limitations, which informs the rational development of safe and effective nanotherapeutic approaches for AD.

## 6. Design and Importance of Nano-Drug Delivery Systems for AD

Nanodrug delivery systems (NDS) are nanoscale platforms designed to precisely deliver therapeutic or diagnostic agents to specific sites, using nanomaterials as carriers. These systems provide significant benefits over traditional drug formulations by enhancing pharmacokinetics, stability, and bioavailability. Owing to their small size, high surface-to-volume ratio, and tunable physicochemical properties, nanocarriers enhance permeation across biological barriers, protect drugs from degradation, and enable controlled or targeted release [73]. Surface functionalization enables the concurrent delivery of multiple therapeutic and imaging agents, supporting multifunctional applications such as targeted therapy, multimodal imaging, and theranostics—particularly relevant for AD. A key aspect of NDS design for AD is their ability to cross the BBB. Nanocarriers, which are much smaller than blood capillaries and cellular structures, can penetrate the BBB through various mechanisms, including endocytosis, transcellular diffusion, receptor-mediated uptake, carrier-mediated transport, and adsorptive-mediated transcytosis [74]. Important physicochemical characteristics—size, shape, surface charge, hydrophobicity, and composition—determine BBB permeability. Functionalization with specific ligands (e.g., transferrin, lactoferrin, or peptides) enhances receptor-mediated uptake. Conversely, attaching positively charged groups promotes adsorptive-mediated transport through electrostatic interactions with the negatively charged endothelial membranes. These strategic modifications significantly improve brain-targeting efficiency, supporting NDS as a promising approach to AD therapeutics [75].

Nanoparticulate drug delivery systems are an innovative approach for directly delivering therapeutic agents into the brain and show great promise for treating various CNS disorders. These nanoscale structures offer several benefits, including protecting medications from enzymatic and chemical degradation, improving their solubility, and facilitating their passage across biological membranes [76]. By delivering drugs precisely to the target site, targeted nanocarriers can reduce systemic side effects and enhance therapeutic efficacy. Drug delivery currently employs a variety of nanoparticulate systems, including polymeric nanoparticles, liposomes, nanoemulsions, dimers, and antibody-linked nanocarriers. Nanoformulations have the potential for imaging and diagnostic applications, in addition to conventional administration routes such as oral, parenteral, topical, vaginal, and rectal [77]. Nanocarriers have proven effective for nasal mucosal vaccination and drug delivery, as they enhance antigen recognition and stability. Lipid- and polymer-based nanocarriers are particularly suitable for nasal administration, enabling direct transport of medications from the nasal cavity to the brain through the olfactory pathway. Overcoming the BBB remains a significant challenge for brain-targeted therapies [78].

**Figure 4:**
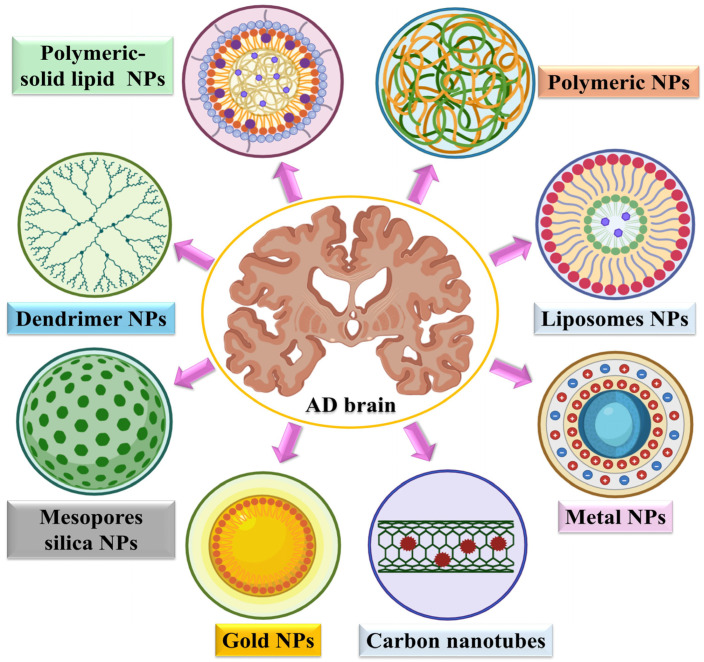
A diagrammatic representation of the primary nanoparticle-based platforms explored for targeted drug delivery in AD. The illustration shows various nanocarrier systems—including polymeric nanoparticles, polymeric–solid lipid nanoparticles, liposomes, metal nanoparticles, dendrimers, mesoporous silica nanoparticles, gold nanoparticles, and carbon nanotubes—developed for targeting and treating AD. These nanomaterials possess a range of physicochemical properties that facilitate enhanced drug encapsulation, improved BBB penetration, targeted delivery to affected areas, and controlled release of therapeutic agents. (Created in BioRender, 2025).

A variety of nanoparticle platforms have been developed to enhance targeted drug delivery for AD, each offering unique structural and functional advantages. Polymeric nanoparticles and polymeric–solid lipid hybrids provide excellent stability, controlled drug release, and high encapsulation efficiency [79]. Liposomes, with their biomimetic lipid bilayers, enhance compatibility and facilitate penetration of the BBB. Metal-based nanoparticles, such as gold and other metals, exhibit therapeutic effects and imaging capabilities due to their optical and catalytic properties [80]. Dendrimers have highly branched structures, making them ideal for precise ligand attachment and multivalent drug binding, while mesoporous silica nanoparticles have tunable pore structures that enable high drug-loading capacity. Carbon nanotubes facilitate efficient cellular uptake and targeted delivery of therapeutic agents. Smart nanomaterial platforms facilitate efficient drug transport across the BBB while enabling precise targeting of Aβ and tau pathologies. By improving BBB penetration, targeting specificity, and controlled drug release, these advanced delivery systems offer promising strategies to enhance therapeutic efficacy and advance the clinical management of AD [40,81] (Figure 4).

Nanoparticulate systems can improve drug transport across the BBB, enabling effective delivery to neural tissues. Developing specialized nasal delivery devices that deliver formulations into the upper nasal cavity, along with surface modifications of nanocarriers, is a key strategy for optimizing nose-to-brain transport [82]. Recent studies have shown successful brain targeting using various nanoparticulate formulations. Lipidic systems include liposomes, transferosomes, solid lipid nanoparticles, and nanostructured lipid carriers, whereas polymeric systems include micelles, nanoparticles, and carbon-based nanovehicles [83]. Additionally, nanogels and nanoemulsions are gaining attention for their efficient transendothelial delivery. The mechanisms of BBB transport—such as receptor-, transporter-, adsorptive-, peptide-, and efflux-mediated pathways—offer potential routes for brain-targeted delivery. Biological therapeutics, including nucleic acids, peptides, and monoclonal antibodies, are under investigation for their neuroprotective effects. Recently, nanovaccines targeting tau proteins involved in AD have shown promise by enhancing antigen stability and immune response, underscoring the potential of nanotechnology-based systems for brain therapies [84,85]. Recent advances in nanomedicine offer effective strategies to overcome the limitations of the BBB through targeted nanoparticles. Smart nanomaterials are engineered to exploit receptor-mediated and adsorptive-mediated transcytosis pathways, enabling efficient BBB crossing and precise drug delivery to the brain, thereby significantly improving targeted drug bioavailability. These nanotechnologies facilitate stage-specific interventions by targeting key AD pathologies, including Aβ aggregation, oxidative stress, and neuroinflammation. Surface functionalization increases targeting specificity, and controlled drug release further enhances therapeutic efficacy [86].

## 7. Strategies for NDS in AD

Current therapeutic strategies for AD prioritize the development of drugs that act centrally within the brain to achieve significant clinical benefits. At present, the FDA has approved only a limited number of medications for AD management, most of which are administered orally, with the exception of rivastigmine, which is also available as a transdermal patch [87]. Oral administration of centrally acting drugs often requires higher doses to reach effective concentrations in the brain due to multiple physiological barriers, including gastrointestinal absorption, first-pass hepatic metabolism, systemic distribution, and the restrictive BBB [88]. This approach frequently results in peripheral adverse effects, such as nausea, vomiting, and diarrhea, which may reduce patient adherence and quality of life. In systemic circulation, unbound drugs generally bind to serum albumin to extend their half-life and maintain stable plasma concentrations, thereby affecting their pharmacokinetic and pharmacodynamic properties [89]. These challenges underscore the necessity for alternative delivery strategies that enhance central nervous system targeting while reducing systemic toxicity [90].

In contrast, nanoparticle-based carriers can maintain prolonged circulation without needing albumin binding. Notably, some nanocarriers can be administered intranasally, bypassing the BBB and enabling direct delivery of therapeutic agents to the brain through the olfactory and trigeminal pathways [91]. This approach not only enhances drug bioavailability at the target site but also decreases systemic side effects. Advances in nanotechnology have revolutionized the treatment of CNS disorders, including AD. Encapsulating drugs in nanocarriers designed for specific targeting significantly improves their accumulation in brain tissue relative to free medicines, owing to enhanced permeability and retention across the BBB. Functionalized nanocarriers with specific surface ligands can further facilitate receptor- or transporter-mediated transcytosis, increasing selectivity for neuronal cells [92]. Additionally, multifunctional nanocarriers have become promising theranostic systems capable of delivering both therapeutic and diagnostic agents simultaneously. These systems can be targeted with specific targeting moieties to bind receptors or transporters at the BBB, thereby enhancing CNS specificity and permeability [93].

The therapeutic complexities of AD stem from its diverse causes and limited access to the brain caused by protective barriers like the BBB and ependymal barrier. Oral medications face additional obstacles, including poor absorption, hepatic metabolism, and rapid clearance, which reduce bioavailability and lead to short half-lives [94]. Many promising therapeutics also suffer from unfavorable physicochemical properties, such as low solubility and instability, hindering their ability to cross the CNS. Nanotechnology-based drug delivery systems have emerged as a promising solution to these issues. Nanocarriers enhance drug stability, regulate drug release, and facilitate effective transport across the BBB via surface modifications and targeting ligands [95]. Their high surface-to-volume ratio enables more efficient site-specific drug delivery and reduces peripheral side effects. This review explores recent advances in polymer-, lipid-, and metal-based nanomaterials used as targeted drug delivery systems for AD, highlighting their ability to increase BBB permeability, improve therapeutic efficacy, and modulate key pathological processes, including Aβ aggregation, tau dysfunction, oxidative stress, and neuroinflammation [96].

### 7.1. Polymeric Nanoparticles for TDD in AD

Polymeric nanoparticles (PNPs) are among the most versatile and widely used nanocarrier systems in nanomedicine owing to their customizable physicochemical properties and ease of manufacture via various synthesis methods. Typically, their size ranges from 10 to 500 nm, enabling PNPs to effectively encapsulate both hydrophilic and hydrophobic therapeutic compounds [97]. The polymeric composition of PNPs, whether natural or synthetic, determines their surface charge, which significantly influences biological behaviors such as mucoadhesion, cellular interactions, and the capacity to traverse biological barriers. Structurally, PNPs are categorized as either nanocapsules, which possess a liquid drug core encased by a polymer shell, or nanospheres, which feature a solid matrix with the drug uniformly dispersed or adsorbed on the surface [98,99]. The FDA-approved polymers polylactide (PLA), poly(lactide-co-glycolide) (PLGA), chitosan, polyethyleneimine (PEI), and poly(ε-caprolactone) (PCL) are frequently utilized for PNPs production [100,101]. These nanocarriers offer several advantages, including high drug-loading capacity, controlled release profiles, customizable surface modifications for targeted brain delivery, biocompatibility, biodegradability, and safe elimination from the body. Nevertheless, challenges persist, particularly regarding the use of organic solvents during synthesis, which may affect formulation safety and scalability. Despite these limitations, PNPs have shown considerable promise in the diagnosis and treatment of neurodegenerative diseases, including AD [102].

Recent research has shown the potential of natural bioactive compounds encapsulated in PNPs for AD therapy. Phuna et al. [103] developed functional PLGA nanoparticles (FNPs) that co-encapsulate curcumin and piperine to address their low solubility and limited bioavailability. The optimized formulation, with an average size of about 116.6 nm, exhibited high encapsulation efficiency and controlled drug release in vitro. Pharmacokinetic studies revealed increased systemic exposure and prolonged circulation time compared to unencapsulated curcumin and piperine. In rat models of AD, systemic delivery of FNPs significantly reduced amyloid accumulation and oxidative stress. It also alleviated neuroinflammation and improved learning and memory functions. Mechanistic studies indicated that co-delivering piperine enhanced neuronal uptake and antioxidant effects. Another study has described the synthesis of chitosan-based polymeric nanoparticles (CSNPs) that encapsulate a decoy peptide (DP) to block the harmful interaction between Aβ and ABAD in AD. The optimized small nanoparticles, approximately 59 nm in size, successfully crossed the BBB and accumulated selectively in the brain. In animal tests, DP-loaded CSNPs improved mitochondrial function by increasing ATP and SOD activity while reducing Aβ levels [104].

Komur et al. developed donepezil-loaded PLGA nanoparticles via a double-emulsion solvent-evaporation method, fine-tuning the PVA concentration and sonication conditions to achieve optimal physicochemical properties. The refined nanoparticles exhibited uniform particle size, high drug-loading capacity, sustained-release profiles, and excellent stability in colloidal form. In vivo pharmacokinetic assessments revealed improved brain accumulation and a longer systemic half-life than traditional oral administration of donepezil. In a mouse model of Aβ induction, treatment with PLGA-encapsulated donepezil significantly decreased acetylcholinesterase activity, reduced Aβ buildup in the hippocampus, and improved memory and learning [105]. In another study, researchers demonstrated the creation of chitosan-coated PLGA nanoparticles (PLGA/chit-NPs) for intranasal delivery of insulin to the brain to treat AD. The mucoadhesive PLGA/chit NPs increased insulin permeability by up to 16-fold and notably enhanced brain bioavailability compared with the insulin solution. This sustained-release system effectively minimized systemic side effects, presenting a promising nanodelivery approach for treating AD via the nose-to-brain route [106]. One study described the development of PEG-functionalized carboxylated multi-walled carbon nanotubes (MWCNT-COOH-PEG) for intranasal delivery of curcumin to improve its brain bioavailability in AD. The optimized nanoformulation demonstrated high entrapment efficiency, prolonged release, and notable anti-apoptotic effects in PC12 cells. Drug delivery significantly increased brain CUR levels, highlighting a promising neuroprotective nanocarrier approach for Alzheimer’s therapy [107].

**Figure 5:**
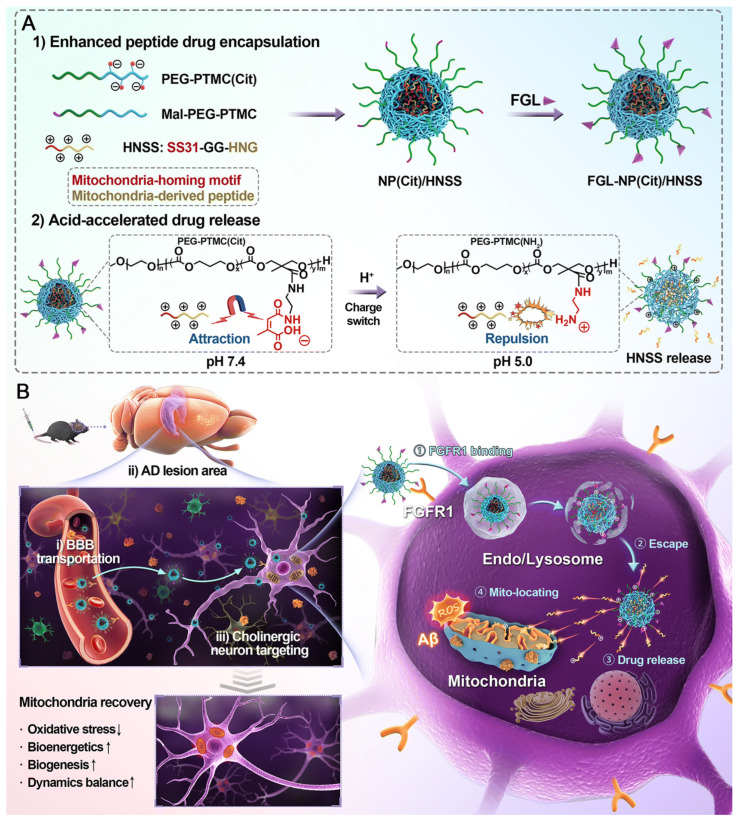
A schematic representation of the nanosystem targeting cholinergic neuronal mitochondria (FGL-NP(Cit)/HNSS) developed to address mitochondrial dysfunction in AD. (**A**) The creation of FGL-NP(Cit)/HNSS illustrates acid-responsive charge reversal and characteristics for drug release. (**B**) In vivo delivery and intracellular mechanism of action: (1) FGL-NP(Cit)/HNSS binds to fibroblast growth factor receptor-1 (FGFR1) on cholinergic neurons; (2) the nanosystem undergoes endocytosis and escapes from endo/lysosomes in the acidic environment; (3) HNSS is released intracellularly; and (4) the released peptide targets mitochondria via the SS31 motif, reducing oxidative stress and restoring mitochondrial function. Adapted from [108] with reproduced permission from ACS Nano (2022), American Chemical Society.

A study described the development of a multifunctional hybrid peptide nanosystem (FGL-NP(Cit)/HNSS) to target mitochondrial dysfunction in AD. The hybrid peptide HNSS, which combines the antioxidant SS31 and the neuroprotective S14G-Humanin, was successfully integrated into an acid-responsive PEG-PTMC(Cit) polymer through electrostatic interactions. The addition of FGL peptides enabled selective targeting of cholinergic neurons that overexpress FGFR1, resulting in a 4.8-fold increase in brain accumulation. The nanosystem exhibited pH-triggered charge reversal, improving lysosomal escape and promoting localization of HNSS within mitochondria. In AD mice models, FGL-NP(Cit)/HNSS decreased Aβ aggregation and tau hyperphosphorylation, increased memory function, and improved mitochondrial function via the PGC-1α and STAT3 pathways (Figure 5). The study presented a promising platform for the targeted delivery of mitochondrial peptides for treating AD and other neurodegenerative disorders [108].

Rivastigmine-loaded PLGA nanoparticles (RIV-PLGA NPs) were created by Imam et al. [109] to increase brain bioavailability and boost treatment efficacy in AD. The nanoparticles were produced by solvent evaporation and nanoprecipitation, showing uniform size, high encapsulation efficiency, sustained drug release, and excellent physiological stability. Intranasal administration of the drug significantly increased brain drug accumulation and improved memory and cognitive abilities in animal models with AD. Kalra et al. [87] developed mucoadhesive rivastigmine nanoparticles (RVT-NPs) and optimized them using both in silico and experimental methods to enhance nasal retention and brain uptake. The formulation had an optimal size, successful mucoadhesion, and a sustained-release profile. When administered intranasally in scopolamine-induced models, it produced notable improvements in cognitive function, increased brain drug levels, and effective acetylcholinesterase inhibition, with minimal nasal toxicity. This research highlighted the potential of mucoadhesive nanoparticles as an effective strategy for delivering cholinesterase inhibitors to the CNS. Another study described the creation of mannose-conjugated, chitosan-coated PLGA nanoparticles (CHTMAN-PLGA) designed for dual delivery of cannabidiol (CBD) and brain-derived neurotrophic factor (BDNF) to treat AD. The addition of mannose enabled GLUT-1-mediated targeting to the brain, and the nanoparticles showed sustained CBD release for up to 22 days. Lab tests revealed that pBDNF transfection efficiency increased 4-fold, resulting in higher BDNF expression in both neuronal and glial cells. The formulation was found to be biocompatible, non-toxic, and hemocompatible, reinforcing its promise as a dual-action nanocarrier for neuroprotection and cognitive recovery in AD [110]. In another investigation, chitosan- and alginate-based nanoparticles loaded with catechin were prepared via ionotropic gelation to address AlCl_3_-induced AD in rats. This nanoformulation significantly restored antioxidant levels, reduced AChE activity, and improved learning and memory, as demonstrated in the Morris water maze test [111].

Zameer et al. [112] described the development of alendronate-loaded chitosan nanoparticles for intranasal brain delivery to manage AD. These nanoparticles, synthesized via ionic gelation, exhibited nanoscale dimensions, low polydispersity, a positive zeta potential, and high drug entrapment efficiency. In an intracerebroventricular streptozotocin (ICV-STZ)-induced AD model, the formulation significantly improved neurobehavioral, neurochemical, and histopathological outcomes, underscoring its promise as a brain-targeted nanotherapeutic strategy for AD. Shahidi et al. [113] demonstrated that combined therapy with MSCs and SeNPs resulted in significant neuroprotective effects in an ICV-STZ-induced rat model of AD. This administration significantly improved cognitive performance in both the novel object recognition and passive avoidance tests compared with monotherapy. Biochemical analyses revealed increased antioxidant capacity, elevated brain-derived neurotrophic factor levels, and reduced amyloid pathology. SeNPs enhance the therapeutic efficacy and survival of transplanted MSCs, supporting the potential of nanoparticle-assisted stem cell therapy as a synergistic approach for AD management. Hashemi-Firouzi et al. [114] demonstrated that PVA-SeNPs produced significant neuroprotective effects in an ICV-STZ-induced rat model of AD. Administration of PVA-SeNPs enhanced cognitive performance in both novel object recognition and passive avoidance learning tasks compared to uncoated SeNPs. Biochemical and histological analyses indicated increased hippocampal brain-derived neurotrophic factor levels, reduced malondialdehyde concentrations, and decreased amyloid-β plaque burden. These findings indicate that surface-modified selenium nanoparticles may mitigate oxidative stress and amyloid pathology, thereby alleviating memory deficits associated with AD.

A comparative analysis evaluated the efficiency of brain delivery of polymeric nanoparticles (PLA-PEG NPs) and extracellular vesicle (EV)-based nanocarriers loaded with donepezil. This medication inhibits acetylcholinesterase for treating AD. Both systems demonstrated advantageous physicochemical properties; however, EV carriers exhibited greater stability, higher blood–brain barrier permeability, and higher neuronal uptake. In vivo results indicated that EV-donepezil significantly improved cognitive function, reduced amyloid-β buildup, and lessened oxidative stress, exceeding the performance of polymeric formulations. While PLA-PEG NPs provided sustained drug release and moderate neuroprotective effects, the study concluded that biologically derived EV nanocarriers may offer greater therapeutic potential for the targeted management of AD [115]. In a separate study, a multifunctional polymeric nanoplatform was developed for nonviral co-delivery of small molecules and nucleic acids to the brain. The PEGylated mucoadhesive carrier improved nasal retention, protected the genetic material, and facilitated targeted delivery [116]. In another study, Auranofin-loaded PLGA-NPs were developed to address AD induced by aluminum chloride. The optimized formulation demonstrated a high entrapment efficiency of 98%, enhanced brain protection, and greater antioxidant and anti-inflammatory effects than free auranofin, indicating a promising polymer-based nanotherapeutic approach for the management of AD [117]. In addition, Handa et al. found that PLGA nanoparticles coated with mannose and co-delivering donepezil and memantine improved brain targeting via intranasal administration, resulting in decreased Aβ accumulation and neuroinflammation while enhancing cognitive function. This dual-drug nanoparticle system employed a synergistic, receptor-mediated strategy to effectively manage AD [118].

### 7.2. Lipid Nanoparticles for TDD in AD

Nanoscale colloidal carriers, known as lipid nanoparticles (LNPs), have shown promise as targeted vehicles for medication delivery in AD. These carriers offer regulated drug release, are low-toxic, highly biocompatible, shield encapsulated medications from deterioration, and enable efficient transport across the BBB [119]. There are two main types of LNPs: solid lipid nanoparticles (SLNs), which have a solid lipid core, and nanostructured lipid carriers (NLCs), which combine liquid and solid lipids to enhance stability and drug-loading capacity. BBB permeability and neuronal targeting are strongly influenced by the physicochemical properties of LNPs, including particle size, zeta potential, and encapsulation efficacy. To improve brain bioavailability and therapeutic efficacy in the management of AD, LNPs have been investigated for the delivery of neuroprotective, anti-amyloid, and antioxidant drugs. Additionally, liposomes—spherical nanovesicles with a bilamellar phospholipid structure—have attracted considerable attention as adaptable drug-delivery systems for AD therapy [120]. Both hydrophilic and lipophilic medications can be encapsulated owing to their amphiphilic nature; hydrophilic agents are contained within the aqueous core, whereas lipophilic compounds are incorporated into the lipid bilayer. This capacity to load both types of medicines simultaneously enables the co-delivery of medications with different solubility properties, thereby improving pharmacokinetic profiles and therapeutic efficacy. Furthermore, liposomes offer biocompatibility, controlled drug release, and the potential for surface modification, making them an attractive choice for improving brain-targeted delivery across the BBB in AD treatment [121].

**Figure 6:**
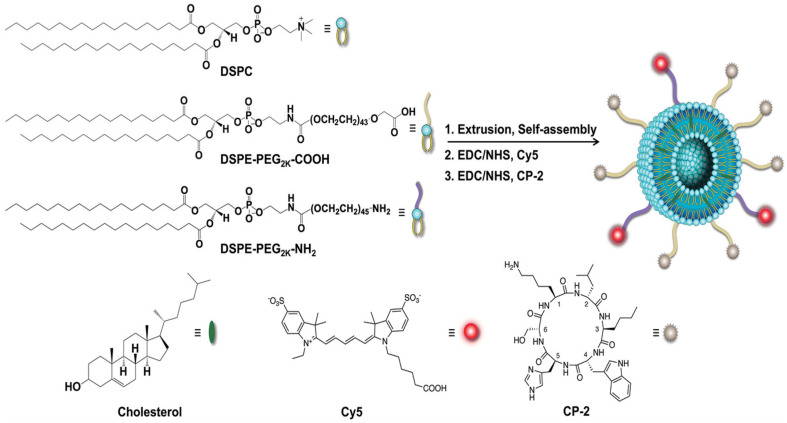
Schematic representation of multifunctional liposomes composed of DSPC, cholesterol, DSPE-PEG_2_k-COOH, and DSPE-PEG_2_k-NH_2_. The liposome surface was functionalized with the oligomer-specific cyclic d,l-α-peptide CP-2 and the fluorescent probe Cy5, enabling early diagnosis and targeted therapy of AD. Adapted from [122] with permission from Wiley-VCH GmbH under the CC BY 4.0 license.

Senapati et al. [122] developed a versatile liposome-based nanoplatform for the early detection and treatment of AD that targets toxic soluble amyloid β oligomers (AβOs). As shown in Figure 6, cyclic d,l-α-peptide (CP-2) and the fluorescent dye Cy5 were attached to liposomes using EDC/NHS chemistry, creating CP-2-LPs with a stable bilayer structure and high biocompatibility. These CP-2-LPs specifically target AβOs, effectively prevent aggregation, and reduce neurotoxicity. In both C. elegans and transgenic AD mouse models, CP-2-LPs enhanced cognitive function and extended lifespan. Additionally, fluorescently labeled CP-2-LPs successfully crossed the BBB, enabling precise brain targeting and dual diagnostic-therapeutic capabilities for AD.

He et al. [123] created felodipine@LND, a liposomal nanodrug that encapsulates the calcium channel blocker felodipine, to help AD-affected neurons regain calcium homeostasis. Felodipine@LND made effective brain delivery possible in murine models by using low-intensity pulsed ultrasound (LIPUS) to transiently open the BBB. This intervention reduced Aβ aggregation, increased mitophagy, suppressed NLRP3 inflammasome activation, and activated the PERK–Nrf2 antioxidant pathway, collectively preventing neuronal cell death. Behavioral assessments showed significant improvements in cognitive function, and histological evaluations revealed reduced amyloid plaques in cortical and hippocampal regions. A recent study revealed a lipid nanoparticle (LNP) system functionalized with lactoferrin (Lf) designed for efficient delivery from the nose to the brain in AD treatment. The LNPs encapsulated α-mangostin (α-M) and BACE1 siRNA (siB) simultaneously to provide dual neuroprotective effects by reducing Aβ production and aiding its clearance. Created using microfluidic techniques, the LNPs displayed uniform size, high encapsulation efficiency, and stability. Nasal delivery enabled effective brain targeting via Lf-mediated transcytosis. In APP/PS1 mice, the treatment reduced Aβ plaques, alleviated neuroinflammation and oxidative stress, and improved cognitive function [124]. Another study described the preparation of cationic nanoliposomes containing artesunate (ART-CLP) through thin-film hydration to enhance ART’s neurotherapeutic effects against AD. The optimized formulation showed a nanoscale size, high encapsulation efficiency, and a controlled release profile. ART-CLP successfully crossed the BBB and significantly inhibited the TLR4/MyD88/NF-κB and NLRP3 inflammasome pathways, reducing Aβ/Tau accumulation, neuroinflammation, and pyroptosis, while improving hippocampal neuronal survival and cognitive function in AD models [125].

Recent research has developed chitosan-coated phosphatidylcholine liposomes to enhance the delivery of donepezil (DZ), a common acetylcholinesterase inhibitor used in the treatment of AD. Designed to bypass the BBB and reduce first-pass metabolism, the optimized nanoparticles (74.86 nm) exhibited high encapsulation efficiency, stability, and biocompatibility up to 62.5 μg/mL. The chitosan coating significantly increased permeability and cellular uptake (66.8 ± 10.6%) in endothelial cells. In vivo studies showed a notable increase in brain DZ accumulation, improved cholinergic neurotransmission, and decreased Aβ deposition in AD mouse models. Behavioral tests revealed substantial improvements in memory and learning, confirming the therapeutic potential of this safe, cost-effective liposomal nanocarrier for the management of AD [126]. Another study explored nanoscavenger techniques, including systems based on high-density lipoprotein (HDL), to address microglial dysfunction in AD. HDL nanocarriers modified with phosphatidic acid and co-loaded with curcumin and a BACE1-targeting siRNA (siBACE1) demonstrated effective BBB penetration and targeted delivery to Aβ plaques. This dual-action approach successfully reduced neuroinflammation, enhanced Aβ clearance, and reversed memory impairments in AD models. Additionally, therapeutic “nanosweeper” designs utilizing neutrophil-mediated transport further improved BBB penetration and Aβ removal efficiency, highlighting HDL-inspired nanoplatforms as promising options for multifunctional AD treatment [127].

**Figure 7:**
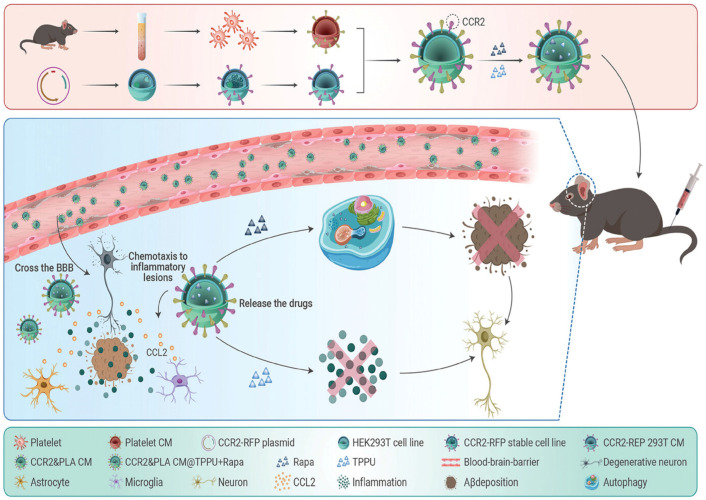
A schematic illustration showing the design and therapeutic mechanism of drug-loaded hybrid cell membrane liposomes for AD treatment. Membranes from platelet cells and HEK293T cells overexpressing CCR2-RFP were fused and simultaneously loaded with rapamycin and TPPU. Following intravenous administration, the nanoliposomes successfully crossed the BBB, targeted neuroinflammatory regions, released therapeutic agents, promoted autophagy, and alleviated AD-associated neuroinflammation. Adapted from [128], Wiley-VCH GmbH under CC BY 4.0 license.

Lin et al. [128] described the development of a hybrid membrane-coated liposomal system for the treatment of AD. The liposomes were coated with fused membranes from platelets and cells overexpressing the chemokine receptor CCR2, which enhances their ability to cross the BBB and target neuroinflammatory lesions specifically. After loading with two synergistic drugs, Rapamycin and TPPU, the hybrid liposomes were administered to transgenic mice, resulting in significant reductions in amyloid-β plaque burden, neuroinflammation, and cognitive impairments (Figure 7). These biomimetic nanosystems with hybrid cell membrane coatings demonstrate improved BBB penetration and multi-targeting capabilities in AD, offering a promising avenue for innovative nanotherapeutic approaches. Another study indicated that lipid-based nanoparticles containing curcumin (LNPs-CUR) effectively enhanced curcumin bioavailability and therapeutic efficacy in AD. In vivo experiments showed that LNPs-CUR decreased amyloid plaque formation, improved cognitive performance, and reduced toxicity compared to free curcumin, underscoring its potential as a nanocarrier for AD treatment [129].

Shan et al. [130] developed a multifunctional liposomal nanocarrier (KLVFF@LIP-CeO_2_) for the combined treatment of AD by targeting Aβ aggregation and oxidative stress. This system simultaneously delivers the Aβ-binding peptide KLVFF and reactive oxygen species (ROS)-neutralizing cerium oxide (CeO_2_) via intranasal administration, thereby enabling effective accumulation in the brain. In HT22 cells, KLVFF@LIP-CeO_2_ inhibited Aβ aggregation, reduced ROS levels, and prevented apoptosis. In APP/PS1 transgenic mice, the treatment significantly reduced Aβ accumulation, alleviated oxidative stress, and improved cognitive function. Zhang et al. [131] reported that liposome-encapsulated Ligustilide (LIG-LPs) effectively reduced oxidative stress and pathological features associated with AD in APPswe/PS1dE9 transgenic mice. Treatment with LIG-LPs notably decreased Aβ accumulation, improved mitochondrial integrity, and restored the balance between mitochondrial fission and fusion. The research showed that LIG-LPs reversed the oxidative stress-induced decline in cAMP-dependent protein kinase A (PKA) and A-kinase anchor protein 1 (AKAP1) signaling, thereby enhancing antioxidant defenses and cognitive function. Additionally, the liposomal encapsulation increased the stability and safety of LIG. Another study described the development of dual-modified nanoliposomes (DPMT@PEI/miR-195) that encapsulate polyethyleneimine/miR-195 complexes to enhance BBB permeability and improve therapeutic outcomes in AD. By being functionalized with mannose and the TAT peptide, these liposomes enhanced miRNA delivery and significantly reduced cognitive decline in APP/PS1 mouse models [132].

A separate study indicated that mitochondrial dysfunction significantly contributes to the progression of AD. To address this, a liposomal formulation of lithospermic acid B (MT-LIP@LA) was developed using D-mannosamine-cholesterol/DSPE-PEG2000-Tet1/lecithin for targeted delivery to neurons. This system increased brain accumulation by 4.3-fold, simultaneously activating mitophagy and mitochondrial biogenesis via the PINK1/LC3B/P62 and PGC-1α/Nrf2 pathways, thereby restoring mitochondrial function and improving cognitive abilities in mice, demonstrating strong neuroprotective effects [133]. To create a transferrin (Tf)-modified liposome encapsulating caffeic acid for enhanced brain targeting in AD treatment. The optimized nanoparticles, approximately 140 nm in size, showed high encapsulation efficiency, sustained CA release for up to 8 days, and excellent stability. In vitro studies demonstrated that CA-loaded Tf-liposomes successfully inhibited Aβ aggregation, disrupted mature fibrils, and reduced amyloid-induced toxicity, establishing them as a promising targeted nanoplatform for the prevention and treatment of AD [134].

Nonetheless, despite their therapeutic potential, several limitations hinder the clinical use of liposomal nanocarriers. A significant challenge is maintaining liposomal stability in physiological environments, as changes in osmolarity, salinity, pH, and temperature can induce aggregation, fusion, or drug leakage, resulting in premature release and reduced effectiveness. Additionally, scaling up liposome production is difficult—laboratory methods such as thin-film hydration and extrusion lack consistency and yield liposomes with varying sizes and lamellarity. Although liposomal nanoparticle-based therapies have been successfully used for various neurodisease, including neurodegenerative diseases, their large-scale production remains a significant challenge. Furthermore, ensuring consistency across batches remains problematic. This can affect the pharmacokinetics, biodistribution, and therapeutic efficacy of the drug and is particularly significant for complex biological drugs, such as nucleic acids and proteins used to treat AD [135].

### 7.3. Metal-Based Nanoparticles for TDD in AD

Due to their unique physicochemical and biological characteristics, inorganic or metal-based nanoparticles, such as gold, silver, and platinum, as well as magnetic nanoparticles, such as superparamagnetic iron oxide nanoparticles, have attracted considerable interest for the treatment of AD [136]. These nanostructures typically range from 1 to 100 nm in size and exhibit high stability, hydrophilicity, biocompatibility, and lower toxicity than organic carriers. Notably, gold nanoparticles have demonstrated significant inhibitory effects on Aβ fibril formation, tau protein aggregation, neurofibrillary tangle development, and acetylcholinesterase activity [137]. Their adjustable size and surface chemistry allow precise functionalization with targeting ligands, peptides, or antibodies to improve BBB penetration and enable site-specific drug delivery. Additionally, magnetic nanoparticles offer dual therapeutic and diagnostic capabilities through magnetic targeting and imaging. Metal-based nanoparticles are promising platforms for targeted DDS in AD, enhancing therapeutic efficacy while reducing off-target effects and boosting BBB transport efficiency [138].

**Figure 8:**
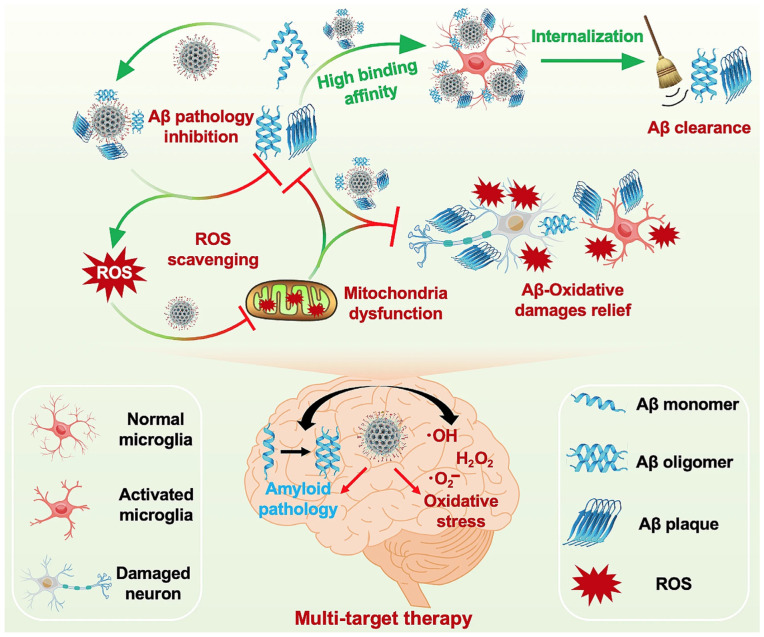
Schematic illustration of RVG29-bMSNs@Ce-1F12 for combination therapy of AD, showing dual targeting of Aβ and ROS to relieve mitochondrial dysfunction and oxidative damage. The nanocomposite promotes Aβ clearance, reduces oxidative stress, and restores neuronal function. Adapted from [139], Journal of Nanobiotechnology, Springer Nature, under CC BY 4.0 license.

Zhang et al. [139] reported that the accumulation of Aβ and oxidative stress are significant contributors to Alzheimer’s disease pathology. To address these concerns, the researchers developed RVG29-bMSNs@Ce-1F12, a dual-targeting nanocomposite comprising biodegradable mesoporous silica nanoparticles conjugated to the Aβ_42_-specific antibody 1F12 and containing ultra-small cerium oxide nanocrystals. The rabies virus glycoprotein peptide (RVG29) was included to facilitate effective BBB penetration and enable targeted administration to brain regions with high Aβ levels. RVG29-bMSNs@Ce-1F12 effectively reduced Aβ misfolding and improved Aβ clearance in APP/PS1 transgenic mice models. It eliminated reactive oxygen species, thereby reducing tau hyperphosphorylation, decreasing neuroinflammation, and improving cognitive function. The schematic illustrates how the multifunctional nanoplatform performs dual actions by inhibiting Aβ aggregation, scavenging ROS, restoring mitochondrial function, and reducing oxidative damage in AD (Figure 8).

Chang et al. [140] detailed the synthesis of biocompatible gold nanoparticles conjugated to cysteine–Aβ peptides (Cys-Aβ@AuNPs), designed as a dual-purpose nanosystem for early detection and prevention of Aβ aggregation in AD. These nanoparticles demonstrated remarkable sensitivity, capable of detecting Aβ peptides in human plasma at sub-femtomolar levels via unique spectral changes associated with Aβ aggregation. Additionally, Cys-Aβ@AuNPs detected early-stage Aβ oligomerization, surpassing traditional thioflavin-T assays, and modulated Aβ aggregation pathways via peptide binding and centrifugation, thereby preventing the formation of harmful oligomers and fibrils. This innovative nanoplatform has significant potential for diagnostic and therapeutic applications in the management of early-stage AD. A separate study reported the synthesis of chiral mSiO_2_ nanospheres via a chiral amide-gel-directed method, thereby introducing molecular-scale chirality into the silica structure. These nanospheres exhibited a high surface area and reduced amyloid-β_42_ aggregation by approximately 79%, significantly decreasing Aβ-induced cytotoxicity in SH-SY5Y cells and highlighting their therapeutic potential for AD [141].

Yin et al. [142] developed a dual-functional nanoinhibitor using functionalized endohedral metallofullerene (f-Gd@C_82_) nanoparticles to prevent and reverse Aβ aggregation related to AD. The addition of hydrogen-bonding and charged surface groups enabled f-Gd@C_82_ to modify Aβ self-assembly, resulting in disordered, non-toxic forms, decreasing protofibril formation, and breaking down mature fibrils. These nanoparticles substantially reduced Aβ-induced neurotoxicity, protected against neuronal death and synaptic loss, demonstrated excellent cytocompatibility, and crossed the BBB. Molecular dynamics simulations confirmed their mechanisms of inhibition. This multifunctional nanoplatform offers a promising approach for AD treatment, focusing on preventing and reversing aggregation while enhancing bioavailability and therapeutic efficacy. Wang et al. [143] reported that glutathione-sensitive silica nanocapsules conjugated to glucose and a rabies virus glycoprotein peptide can effectively deliver CRISPR genome editors throughout the body, including across the BBB. In vivo studies demonstrated successful neuronal modifications, achieving up to 28% editing of Cre mRNA and notable reductions in APP and TH expression. Noor et al. [144] demonstrated that curcumin NPs exerted significant neuroprotective effects in an ICV-STZ-induced rat model of AD. The findings indicated a reduction in oxidative stress, as evidenced by decreased lipid peroxidation and nitric oxide levels, along with restoration of reduced glutathione levels in the cortex and hippocampus. Furthermore, curcumin nanoparticles normalized AChE activity, inflammatory markers, Na^+^/K^+^-ATPase activity, and neurotransmitter levels.

Liu et al. [145] reported the development of human serum albumin (HSA)-incorporated ultrasmall copper nanoclusters (CuNCs@HSA) as a versatile nanotherapeutic for AD. The CuNCs@HSA exhibited remarkable enzyme-mimetic antioxidant capabilities, including functions similar to those of superoxide dismutase, catalase, and glutathione peroxidase, thereby effectively neutralizing reactive oxygen species. They were significantly more effective—2.5 times more effective than native HSA—in inhibiting Aβ fibrillization and reducing neuroinflammation by decreasing TNF-α and IL-6 secretion. In vitro experiments showed that CuNCs@HSA reduced Aβ-induced cytotoxicity. In contrast, in vivo studies indicated that they prevented plaque formation, reduced oxidative stress, and extended lifespan in transgenic C. elegans, highlighting their strong potential as combined antioxidant and anti-amyloid therapies for AD. Yin et al. [146] described the creation of ultra-small carbon nitride nanodots (C3N) that serve as effective inhibitors of Aβ aggregation in AD. These nanodots successfully blocked Aβ-induced neuronal damage, restored synaptic function, and decreased fibrillar plaque buildup in APP/PS1 mice. Molecular dynamics simulations showed that C3N nanodots interfere with Aβ aggregation pathways, and in vivo studies demonstrated their high biocompatibility and neuroprotective abilities for AD treatment.

Yin et al. [147] stated that existing treatments for AD mainly target Aβ aggregation and do not address the resulting oxidative stress and neuronal cell death. They developed K8@Fe–Rh/Pda nanoparticles using a stepwise metal–phenolic coordination strategy that combines rhein with polydopamine. Polydopamine prevented Aβ oligomer aggregation via catechol, imine, and π-π interactions, whereas rhein facilitated repair of neuronal damage. The nanoparticles activated the SIRT1/PGC-1α signaling pathway, promoting mitochondrial biogenesis and reducing oxidative damage. Yang et al. [148] developed selenium nanoparticles (Tg-CS/DMY@SeNPs) that cross the blood–brain barrier and are coated with chitosan and dihydromyricetin (DMY) to reduce neuroinflammation in AD. These multifunctional nanoparticles successfully crossed the BBB, inhibited Aβ aggregation, and decreased inflammatory cytokine secretion by modulating the NF-κB pathway in APP/PS1 mice. Additionally, Tg-CS/DMY@SeNPs improved gut barrier integrity and altered the gut microbiota, particularly increasing Gordonibacter abundance, thereby downregulating NLRP3 inflammasome expression. This interaction among gut microbes, the NLRP3 inflammasome, and the brain enabled Tg-CS/DMY@SeNPs to significantly reduce neuroinflammation and oxidative stress. Ruan et al. [149] described the creation of a multifunctional nanotheranostic platform composed of curcumin-loaded superparamagnetic iron oxide nanoparticles (SPIO) encapsulated with DSPE-PEG and modified with CRT and QSH peptides. This technology enabled precise MRI detection and quantification of β-amyloid plaques in APP/PS1 mice and notably reduced Aβ accumulation and memory impairments by blocking NLRP3 inflammasome activation, providing both diagnostic and therapeutic options for AD.

Redox-active metal ions, especially Cu^2+^, are vital in the progression of AD by causing oxidative stress and promoting the formation of harmful Cu^2+^–Aβ aggregates. Managing metal ion balance is thus considered a potentially practical therapeutic approach. Li et al. [150] developed silicon–carbon dots (SiCDs) using amino-containing silane and the disodium salt of ethylenediaminetetraacetic acid as dual carbon sources to achieve efficient Cu^2+^ chelation. To enhance localized concentration and chelating ability, SiCDs were incorporated into mesoporous silica nanoparticles (mSiO_2_) through silane–silanol interactions, producing mSiO_2_@SiCDs. These nanocomposites specifically chelated Cu^2+^, inhibited Cu^2+^-driven Aβ aggregation, reduced oxidative stress, and displayed anti-inflammatory and neuroprotective effects both in vitro and in *C. elegans*, highlighting their potential as a treatment for AD. Ge et al. [151] created multifunctional KLVFF@Au–CeO_2_ (K-CAC) nanocomposites designed for AD therapy. Ceria nanoparticles (CeO_2_NPs) exhibited catalase and superoxide dismutase mimetic activities, thereby decreasing oxidative stress. Meanwhile, the gold nanorods (Au NRs) enabled near-infrared (NIR)-induced photocatalytic and photothermal effects. The arrangement of the CeO_2_–Au structure improved catalytic efficiency and increased permeability across the BBB. Additionally, the Aβ-targeting KLVFF peptides enhanced selective binding and therapeutic efficacy, showing strong neuroprotective and anti-amyloid effects both in vitro and in vivo, thus demonstrating significant potential for AD treatment.

**Figure 9:**
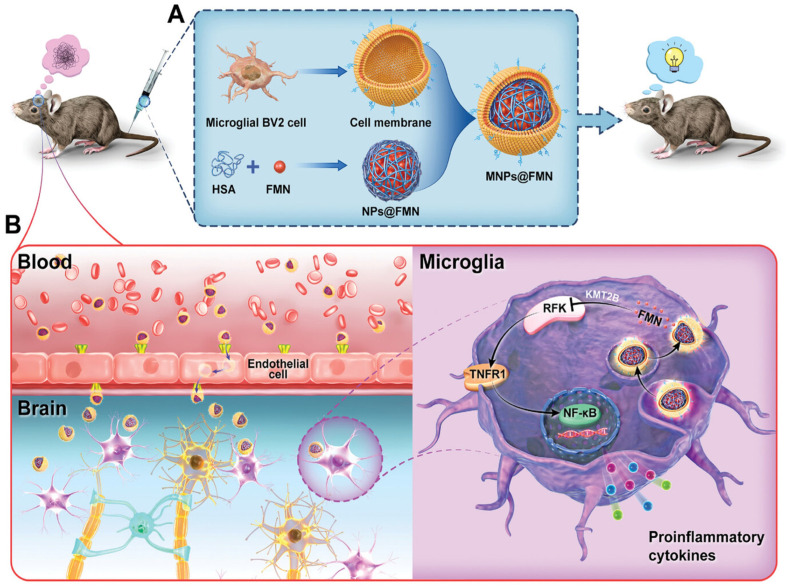
(**A**) Preparation of microglia membrane–coated nanoparticles (MNPs@FMN). FMN-loaded nanoparticles (NPs@FMN) are first constructed using human serum albumin (HSA), followed by coating with BV2 microglial cell membranes to generate biomimetic MNPs@FMN, which enhance biological stability and targeting capability in vivo. (**B**) In vivo transport and anti-neuroinflammatory mechanism of MNPs@FMN. After systemic administration, MNPs@FMN circulate in the bloodstream, cross the BBB, and selectively accumulate in the brain. The nanoparticles preferentially target microglia, where FMN is released intracellularly to inhibit riboflavin kinase (RFK) through KMT2B regulation. This inhibition suppresses TNFR1/NF-κB signaling, reduces proinflammatory cytokine production, alleviates neuroinflammation, and ultimately restores cognitive function. Adapted from [153], Advanced Science, Wiley-VCH GmbH under the provisions of the CC BY 4.0 license.

Zhang et al. [152] described the green synthesis of AgNPs and assessed their therapeutic efficacy in a rat model of sporadic AD induced by ICV-STZ. The biosynthesized AgNPs displayed a face-centered cubic crystalline structure and were capped with polyphenols derived from plants. Behavioral assessments, including the Barnes maze and object recognition tests, indicated that AgNPs treatment significantly ameliorated STZ-induced deficits in spatial learning and recognition memory. Zhang et al. [153] demonstrated that riboflavin kinase (RFK), an essential enzyme in riboflavin metabolism, is predominantly expressed in microglia and contributes to neuroinflammation-associated cognitive impairment. Their findings indicate that flavin mononucleotide (FMN) downregulates RFK expression by modulating lysine-specific methyltransferase 2B (KMT2B), which, in turn, suppresses TNFR1/NF-κB signaling. Furthermore, biomimetic microglial nanoparticles loaded with FMN (MNPs@FMN) effectively traversed the blood–brain barrier and attenuated neuroinflammation. These nanoparticles also enhanced cognitive and synaptic functions in lipopolysaccharide-induced mouse models of AD, underscoring the therapeutic potential of FMN for inflammation-mediated neurodegeneration (Figure 9).

A study by Ren et al. [3] reported mitochondria-targeted nanozymes, specifically bromide-conjugated MoS_2_ quantum dots (TPP-MoS_2_ QDs), for the treatment of AD. These nanozymes successfully crossed the BBB, targeted the mitochondria, and reduced Aβ-induced neurotoxicity by shifting microglial polarization from the proinflammatory M1 phenotype to the anti-inflammatory M2 phenotype. Additionally, TPP-MoS_2_ QDs reduced oxidative stress, modulated inflammatory cytokines, and enhanced Aβ aggregate clearance, demonstrating a promising mitochondria-targeted strategy for reducing neuroinflammation and AD-related pathology. A separate study by Li et al. [154] developed a resveratrol-selenium-peptide nanocomposite (TGN-Res@SeNPs) to increase resveratrol bioavailability and enhance its therapeutic effects in AD treatment. The TGN-modified SeNPs effectively crossed the BBB, prevented Aβ aggregation, and alleviated Aβ-induced oxidative stress and neuroinflammation by regulating the NF-κB/MAPK/Akt signaling pathways. Moreover, oral delivery of TGN-Res@SeNPs restored gut microbiota balance and improved cognitive function in AD model mice.

Polymer, lipid, and metal-based nanomaterials have become promising nanocarriers for targeted therapy in AD because they can cross the BBB and deliver therapeutic agents directly to the brain (Table 1). Polymeric NPs, such as PLGA and chitosan, provide controlled release, compatibility with biological systems, and surface modifications for ligand-mediated targeting; however, challenges include limited drug-loading capacity and slow degradation rates. Lipid-based NPs, like solid lipid nanoparticles and nanostructured lipid carriers (NLCs), enhance BBB permeability and protect bioactive compounds. However, they can experience drug leakage and may be unstable over time. Metal NPs offer exceptional imaging and therapeutic capabilities but carry risks of neurotoxicity and oxidative stress due to metal buildup. Despite these obstacles, adding multifunctional coatings, biodegradable materials, and stimuli-responsive designs could improve biocompatibility, targeting accuracy, and therapeutic effectiveness, making these nanocarriers promising candidates for next-generation AD treatments [155].

## 8. Clinical Trials, Limitations, and Challenges in Drug Delivery for AD

Nanotechnology-based drug delivery systems (NTDDS) show great promise in improving the precision and efficiency of delivering therapies to the brain. However, from a clinical neuroscience standpoint, several key issues must be addressed before these systems can proceed to human trials [156]. Primary concerns include whether nanocarriers can reliably be taken up by olfactory sensory neurons for effective transport from the nose to the brain and whether they are safe for long-term use. Most of the current evidence supporting the effectiveness of NTDDS comes from studies in rodents and other non-primate models, which, despite their usefulness, do not fully replicate human neuroanatomy or pharmacokinetics. Consequently, there are significant uncertainties regarding their clinical application, toxicity, and therapeutic success [157,158].

Cochrane reviews have shown that traditional acetylcholinesterase inhibitors produce measurable cognitive improvements in individuals with mild-to-moderate AD [159]. Similarly, extensive randomized trials indicate that memantine enhances cognition and functional outcomes in those with moderate to severe AD, although its effectiveness in earlier stages of the disease is limited. Notably, these results are based solely on oral formulations, highlighting opportunities for NTDDS to enhance drug bioavailability and provide extended-release. Additionally, natural substances such as Huperzine A have been studied within the context of nanotechnology [160]. However, the current trials—mainly small, short-term studies from China—lack sufficient evidence of long-term safety or significant clinical benefits. More comprehensive, long-term, and translational research is necessary before such therapies can be adopted in clinical practice.

Although smart nanoparticle-based strategies for AD demonstrate considerable potential, several limitations impede their clinical translation. Many nanocarriers have limited and inconsistent ability to cross the BBB. Long-term safety concerns, including nanoparticle accumulation, neurotoxicity, and immunogenicity, remain unresolved [161]. Variability in nanoparticle size, surface characteristics, and stability can compromise reproducibility and therapeutic efficacy. Furthermore, challenges in large-scale manufacturing, quality control, and batch-to-batch consistency persist. The absence of standardized regulatory guidelines and the limited availability of clinical trial data further hinder regulatory approval and clinical adoption. Overcoming these challenges is critical to advancing nanoparticle-based therapies for AD [63].

The current treatment for AD mainly relies on FDA-approved medications, with etanercept, a tumor necrosis factor-α inhibitor, sometimes used off-label. Most of these drugs are taken orally in tablet, capsule, solution, or orally dissolving forms. Additionally, rivastigmine is available as a transdermal patch that provides sustained-release delivery, whereas etanercept is administered subcutaneously [162]. Despite these delivery options, effectively transporting therapeutic agents across the highly selective BBB remains a significant challenge, limiting clinical effectiveness and complicating drug development for AD. Drug delivery has emerged as a promising, noninvasive method that uses the olfactory and trigeminal pathways to bypass the BBB and deliver drugs directly from the nose to the brain [163]. Recent preclinical and clinical research indicates improved brain targeting and greater patient compliance than intravenous or oral administration, particularly for long-term treatments. The development of nanocarrier systems has attracted considerable attention; however, their optimization depends heavily on appropriate animal models. Comparative anatomical studies indicate that rabbits closely resemble humans in nasal cavity structure, particularly in mucosal composition and hair follicle distribution, making them useful for translational research involving drug delivery. In contrast, rats have unique anatomical features, such as the forward position of the ciliated respiratory epithelium, and their larger olfactory region limits the direct transferability of findings to humans [164]. Nonetheless, rodent, rabbit, and even nonhuman primate models provide valuable insights into drug deposition, mucociliary clearance, and neural transport processes. Another clinical challenge involves side effects associated with long-term pharmacological treatment for AD. Gastrointestinal problems—such as nausea, vomiting, diarrhea, weight loss, and anorexia—are common, especially with oral AChEIs [165]. These issues highlight the need for alternative delivery methods, including extended-release formulations, sublingual and intranasal systems, transdermal patches, intramuscular injections, and nanotechnology-based targeted DDS. These approaches could improve therapeutic outcomes, reduce systemic side effects, and enhance overall patient compliance and caregiver ease of care.

The clinical translation of nanoparticle-based therapies for AD necessitates thorough consideration of regulatory requirements concerning safety, efficacy, and quality control. Regulatory agencies, such as the FDA, mandate a comprehensive evaluation of nanomaterial properties, including biodistribution, toxicity, immunogenicity, and long-term safety. Standardizing nanoparticle synthesis, ensuring reproducibility, and maintaining batch-to-batch consistency present significant regulatory challenges, as even minor variations in size, shape, or surface characteristics can substantially alter biological behavior [166]. In the field of nanomedicine, regulatory scrutiny is intensified by nanoparticles’ unique interactions with biological systems, their potential accumulation in organs such as the brain, liver, and spleen, and their size- and surface-dependent toxicity profiles. Moreover, the lack of fully harmonized regulatory frameworks specifically tailored for nanotherapeutics complicates approval processes and clinical translation. Overcoming these challenges requires rigorous preclinical assessment, adherence to ethical manufacturing practices, and the implementation of well-designed clinical trials to ensure the safe and effective application of nanoparticle-based therapies for AD [167].

## 9. Conclusions and Future Perspectives

In conclusion, AD remains a significant global health challenge. Current pharmacological therapies, including acetylcholinesterase inhibitors and the NMDA receptor antagonist memantine, offer only limited symptomatic relief and do not alter disease progression. The limited efficacy of these traditional oral treatments underscores the urgent need for an advanced DDS capable of overcoming the BBB. Innovative DDS, particularly those utilizing smart nanoparticles, demonstrate improved stability, targeted delivery, and enhanced brain penetration. The capacity of these systems to modify release profiles and direct therapeutics to affected brain regions underscores their potential to advance future treatment strategies for AD.

Nanotechnology-based drug delivery systems are an emerging platform for enhancing therapeutic precision, bioavailability, and brain targeting. Among these, PNPs provide controlled release, chemical stability, and the ability to deliver both hydrophilic and hydrophobic drugs. Lipid-based nanocarriers improve BBB penetration due to their biomimetic composition and have demonstrated higher encapsulation efficiency for small molecules, peptides, and natural compounds such as Huperzine A. Metal-based nanoparticles, including gold, cerium oxide, and iron oxide systems, offer additional benefits, including antioxidant properties, imaging capabilities, and multifunctional theranostic applications. Together, these nanoplatforms provide versatile ways to target amyloid aggregation, tau pathology, oxidative stress, neuroinflammation, and synaptic dysfunction—key features of AD. Despite these advancements, several scientific challenges hinder clinical use. Significant uncertainties remain regarding the effectiveness of nose-to-brain transport, long-term biocompatibility, and the safety of repeated nanoparticle exposure. Most efficacy data come from rodent and small-animal studies, which do not accurately reflect human neuroanatomy, pharmacokinetics, or the complexities of the BBB. Therefore, further comprehensive translational research, extended toxicity evaluations, and standardized pharmacokinetic analyses are necessary for the application of polymeric, lipidic, and metal-based nanoparticles in the treatment of human AD.

Production and regulatory hurdles further complicate the development process. Ensuring reproducible large-scale manufacturing with consistent physicochemical characteristics—such as particle size, surface charge, morphology, and drug-loading efficiency—is particularly difficult for complex polymeric and lipidic formulations. Metal-based nanoparticles need rigorous control over their composition, oxidation state, and surface chemistry to avoid toxicity. Regulatory agencies currently lack unified standards for assessing nanomedicines, resulting in uncertainty in the approval process. Thorough characterization of nanoparticle biodistribution, stability, biodegradation, and long-term safety is increasingly required, necessitating specialized analytical techniques and disease-specific clinical endpoints. Looking ahead, meaningful advancements will depend on integrating cutting-edge material engineering, detailed mechanistic investigations, and coordinated regulatory strategies. Collaborative efforts among neuroscientists, chemists, clinicians, industry stakeholders, and regulatory bodies are crucial for developing standardized testing protocols and expediting translation. Well-structured preclinical and clinical trials must systematically evaluate toxicity, refine targeting strategies, and confirm therapeutic efficacy across nanoparticle platforms.

Smart nanomaterials play a central role in biosensor development by enhancing biorecognition efficiency, signal transduction, and sensitivity, while also enabling multifunctional theranostic platforms for the diagnosis and treatment of AD. Smart nanomaterial-based delivery systems have emerged as a promising approach to address the significant therapeutic challenges in AD, particularly the BBB’s limited permeability. Nanoparticles made from polymers, lipids, and metals offer versatile physicochemical features—such as size, surface charge, and functionalization—that improve BBB crossing through mechanisms like receptor-mediated transport, adsorptive-mediated transcytosis, and temporary tightening of tight junctions. After crossing the blood–brain barrier, targeted nanoparticles can specifically bind to AD-related biomarkers, such as Aβ plaques, tau clumps, or regions of neuroinflammation, thereby increasing drug accumulation at affected sites and reducing systemic toxicity. These systems also enable controlled or sustained drug release, thereby enhancing therapeutic efficacy compared with traditional approaches. Additionally, incorporating imaging agents into nanoparticle systems enables theranostic applications, supporting real-time tracking of delivery and treatment outcomes. In conclusion, advanced nanotechnologies hold significant potential to improve AD management and support the development of disease-modifying treatments.

## Figures and Tables

**Table 1:** Overview of various nanomaterial platform systems utilized for targeted therapy in AD. The table details each formulation’s therapeutic payload, primary AD-related pathological targets, functional characteristics, suggested or observed signaling mechanisms, and established therapeutic effectiveness in preclinical studies.

S. No.	NPs/NCs Type	Loaded Drug/Agent	AD Target	Functions	Mechanism	Therapeutic Effects	References
1.	Polymeric NPs (PLGA)	Rivastigmine	AChE deficit	Brain delivery, sustained release	Improved AChE inhibition	Better cognition	[109]
2.	Extracellular vesicles	Donepezil	AChE	BBB crossing, targeted	Enhanced AChE inhibition	Improved cognition	[115]
3.	PLGA	Curcumin + Piperine	Aβ, oxidative stress	Antioxidant, anti-amyloid	Improved brain uptake	Cognitive improvement	[103]
4.	PLGA	Donepezil HCl	Cholinergic	Controlled release	Higher brain exposure	Enhanced cognitive effects	[105]
5.	Mucoadhesive polymeric NP	Rivastigmine	Cholinergic	Nasal delivery	Better CNS PK/PD	Improved cognition	[87]
6.	MWCNT (PEG)	Curcumin	Aβ, oxidative stress	Antioxidant delivery	Enhanced uptake	Neuroprotection	[107]
7.	PLGA/chitosan	Insulin	Insulin signaling	Nose-brain delivery	Restored signaling	Cognitive improvement	[106]
8.	Mannose-CS-PLGA	CBD + BDNF	Neuroinflammation	Targeted neurotrophic	BDNF signaling	Reduced inflammation	[110]
9.	PLGA	Auranofin	Oxidative stress	Neuroprotective	Anti-inflammatory	Neuroprotection	[117]
10.	Mannosylated PLGA	Donepezil + Memantine	Cholinergic + NMDA	Combination delivery	Dual mechanism	Cognitive improvement	[118]
11.	Chitosan–Alginate	Catechin	Oxidative stress	Antioxidant	Reduced ROS	Improved cognition	[111]
12.	Liposomal Nanodrug	Felodipine	Mitochondria	BBB-crossing	Mitochondrial restoration	Cognitive benefit	[123]
13.	Lipid NPs	Lactoferrin	Aβ	Nanoscavenging	Aβ clearance	Reduced plaques	[124]
14.	Cationic lipid NPs	Artesunate	Inflammasome	Anti-inflammatory	NLRP3 inhibition	Neuroprotection	[125]
15.	Chitosan liposomes	Donepezil	AChE	Sustained nasal	Improved targeting	Behavioral improvement	[126]
16.	Lipid-like scavenger	Curcumin	Microglia/Aβ	Anti-inflammatory	Modulates microglia	Neuroprotection	[127]
17.	Liposomes+CeO_2_	KLVFF	Aβ + ROS	Dual-target	Peptide binding + ROS scavenging	Reduced plaques	[130]
18.	Liposome	Ligustilide	Aβ_42_ + ROS	Anti-amyloid	ROS + Aβ inhibition	Behavior improvement	[131]
19.	Mesoporous silica	Si–C dots	Cu^2+^/Aβ	Chelation	Inhibits Cu-Aβ oxidative stress	Reduced pathology	[150]
20.	Gold–CeO_2_ hybrid	Plasmonic system	Oxidative stress	Photothermal + antioxidant	Reduced ROS	Neuroprotective	[151]

## Data Availability

Data are available from the authors upon request.

## References

[1] Sighencea M.G., Popescu R.Ș., Trifu S.C. (2024). From fundamentals to innovation in Alzheimer’s disease: Molecular findings and revolutionary therapies. Int. J. Mol. Sci..

[2] Twiss E., McPherson C., Weaver D.F. (2025). Global Diseases Deserve Global Solutions: Alzheimer’s Disease. Neurol. Int..

[3] Ren C., Li D., Zhou Q., Hu X. (2020). Mitochondria-targeted TPP-MoS_2_ with dual enzyme activity provides efficient neuroprotection through M1/M2 microglial polarization in an Alzheimer’s disease model. Biomaterials.

[4] Fanlo-Ucar H., Picón-Pagès P., Herrera-Fernández V., Ill-Raga G., Muñoz F.J. (2024). The dual role of amyloid Beta-Peptide in oxidative stress and inflammation: Unveiling their connections in Alzheimer’s disease etiopathology. Antioxidants.

[5] Di Caro V., Williams C., North H.A., Hamby M.E. (2025). Biomarkers in CNS drug discovery, drug development, and clinical implementation. Trends in CNS Drug Discovery.

[6] Almeida Z.L., Vaz D.C., Brito R.M. (2025). Morphological and molecular profiling of amyloid-β species in Alzheimer’s pathogenesis. Mol. Neurobiol..

[7] Cardillo M., Katam K., Suravajhala P. (2025). Advancements in multi-omics research to address challenges in Alzheimer’s disease: A systems biology approach utilizing molecular biomarkers and innovative strategies. Front. Aging Neurosci..

[8] Ferrari I., Limiti E., Giannitelli S.M., Trombetta M., Rainer A., D’Amelio M., La Barbera L., Gori M. (2025). Microfluidic-Based Technologies for Crossing the Blood–Brain Barrier Against Alzheimer’s Disease: Novel Strategies and Challenges. Int. J. Mol. Sci..

[9] Julius A., Renuka R.R., Pothireddy R.B. (2024). Multi-target drugs to control the progression of age-related neurodegenerative disorders. Aging Med. Healthc..

[10] Kotarba S., Kozłowska M., Scios M., Saramowicz K., Barczuk J., Granek Z., Siwecka N., Wiese W., Golberg M., Galita G. (2024). Potential Mechanisms of Tunneling Nanotube Formation and Their Role in Pathology Spread in Alzheimer’s Disease and Other Proteinopathies. Int. J. Mol. Sci..

[11] Yu P., Shen L., Tang L. (2025). Multimodal DTI-ALPS and Hippocampal Microstructural Signatures Unveil Stage-Specific Pathways in Alzheimer’s Disease Progression. Front. Aging Neurosci..

[12] Alqudah A., Aljabali A.A., Gammoh O., Tambuwala M.M. (2024). Advancements in neurotherapeutics: Nanoparticles overcoming the blood–brain barrier for precise CNS targeting. J. Nanoparticle Res..

[13] Abduljawad A.A., Alkinani K.B., Zaakan A., AlGhamdi A.S., Hamdoon A.A.E., Alshanbari B.H., Alshehri A.A., Alluhaybi B.B., Alqashi S.O.I., Abduljawad R.A. (2025). Targeting Amyloid-β Proteins as Potential Alzheimer’s Disease Therapeutics: Anti-Amyloid Drug Discovery, Emerging Therapeutics, Clinical Trials and Implications for Public Health. Pharmaceuticals.

[14] Benjamin S.R., Lima F.D., Nunes P.I.G., Dutra R.F., Andrade G.M.D., Oriá R.B. (2025). Advanced Biosensing Technologies: Leading Innovations in Alzheimer’s Disease Diagnosis. Chemosensors.

[15] Joshi K.M., Salve S., Dhanwade D., Chavhan M., Jagtap S., Shinde M., Holkar R., Patil R., Chabukswar V. (2025). Advancing Protein Biosensors: Redefining Detection through Innovations in Materials, Mechanisms, and Applications for Precision Medicine and Global Diagnostics. RSC Adv..

[16] Razavi R., Khajouei G., Divsalar F., Dawi E., Amiri M. (2025). Recent advances on brain drug delivery via nanoparticles: Alternative future materials for neuroscience applications; a review. Rev. Neurosci..

[17] Jang Y.J., Kang S.J., Park H.S., Lee D.H., Kim J.H., Kim J.E., Kim D.I., Chung C.H., Yoon J.K., Bhang S.H. (2025). Drug delivery strategies with lipid-based nanoparticles for Alzheimer’s disease treatment. J. Nanobiotechnol..

[18] Dong N., Ali-Khiavi P., Ghavamikia N., Pakmehr S., Sotoudegan F., Hjazi A., Gargari M.K., Gargari H.K., Behnamrad P., Rajabi M. (2025). Nanomedicine in the Treatment of Alzheimer’s Disease: Bypassing the Blood–Brain Barrier with Cutting-Edge Nanotechnology. Neurol. Sci..

[19] Lanoiselée H.M., Nicolas G., Wallon D., Rovelet-Lecrux A., Lacour M., Rousseau S., Richard A.C., Pasquier F., Rollin-Sillaire A., Martinaud O. (2017). APP, PSEN1, and PSEN2 Mutations in Early-Onset Alzheimer Disease: A Genetic Screening Study of Familial and Sporadic Cases. PLoS Med..

[20] Dhapola R., Sharma P., Kumari S., Bhatti J.S., HariKrishnaReddy D. (2024). Environmental toxins and Alzheimer’s disease: A comprehensive analysis of pathogenic mechanisms and therapeutic modulation. Mol. Neurobiol..

[21] Yan Q., Qin Q., Zhang S., Chen F., Ru Y., Zhong Y., Wu G. (2025). Glial Cell Nutrient Sensing: Mechanisms of Nutrients Regulating Alzheimer’s Pathogenesis and Precision Intervention. Crit. Rev. Food Sci. Nutr..

[22] Contador I., Buch-Vicente B., Del Ser T., Llamas-Velasco S., Villarejo-Galende A., Benito-León J., Bermejo-Pareja F. (2024). Charting Alzheimer’s disease and dementia: Epidemiological insights, risk factors and prevention pathways. J. Clin. Med..

[23] Padmanaban A., Taarika G., Geetha R.V., Royapuram P. (2025). Attenuation of oxidative stress and anti-Alzheimer effect of ursolic acid. Texila Int. J. Public Health.

[24] Daly T. (2024). A philosophy of science approach to the amyloid hypothesis of Alzheimer’s disease. Eur. J. Neurosci..

[25] Rajkumar M., Vimala K., Tamiliniyan D.D., Thangaraj R., Jaganathan R., Kumaradhas P., Kannan S. (2022). Gelatin/Polyvinyl Alcohol-Loaded Magnesium Hydroxide Nanocomposite Attenuates Neurotoxicity and Oxidative Stress in Alzheimer’s Disease-Induced Rats. Int. J. Biol. Macromol..

[26] Tondo G., De Marchi F., Bonardi F., Menegon F., Verrini G., Aprile D., Anselmi M., Mazzini L., Comi C. (2024). Novel therapeutic strategies in Alzheimer’s disease: Pitfalls and challenges of anti-amyloid therapies and beyond. J. Clin. Med..

[27] Shin M.K., Schuck A., Kang M., Kim Y.S. (2024). Electrochemical Analysis of Amyloid Plaques and ApoE4 with Chitosan-Coated Gold Nanostars for Alzheimer’s Detection. Biosensors.

[28] Patwekar M., Patwekar F., Khan S., Sharma R., Kumar D. (2024). Navigating the Alzheimer’s Treatment Landscape: Unraveling Amyloid-beta Complexities and Pioneering Precision Medicine Approaches. Curr. Top. Med. Chem..

[29] Jiang G., Xie G., Li X., Xiong J. (2025). Cytoskeletal Proteins and Alzheimer’s Disease Pathogenesis: Focusing on the Interplay with Tau Pathology. Biomolecules.

[30] Di Lorenzo D. (2024). Tau Protein and Tauopathies: Exploring Tau Protein—Protein and Microtubule Interactions, Cross-Interactions and Therapeutic Strategies. ChemMedChem.

[31] Grewal A., Raikundalia S., Zaia J., Sethi M.K. (2025). Overview of Proteomic Analysis of Amyloid Plaques and Neurofibrillary Tangles in Alzheimer’s Disease. Biomolecules.

[32] Abukuri D.N. (2024). Novel biomarkers for Alzheimer’s disease: Plasma neurofilament light and cerebrospinal fluid. Int. J. Alzheimer’s Dis..

[33] Nasb M., Tao W., Chen N. (2024). Alzheimer’s disease puzzle: Delving into pathogenesis hypotheses. Aging Dis..

[34] Tsimpili H., Zoidis G. (2025). A New Era of Muscarinic Acetylcholine Receptor Modulators in Neurological Diseases, Cancer and Drug Abuse. Pharmaceuticals.

[35] Singh R., Panghal A., Jadhav K., Thakur A., Verma R.K., Singh C., Goyal M., Kumar J., Namdeo A.G. (2024). Recent advances in targeting transition metals (Copper, Iron, and Zinc) in Alzheimer’s disease. Mol. Neurobiol..

[36] Rajkumar M., Sakthivel M., Senthilkumar K., Thangaraj R., Kannan S. (2022). Galantamine-Tethered Hydrogel as a Novel Therapeutic Target for Streptozotocin-Induced Alzheimer’s Disease in Wistar Rats. Curr. Res. Pharmacol. Drug Discov..

[37] Mazur T., Malik M., Bieńko D.C. (2024). The impact of chelating compounds on Cu^2+^, Fe^2+/3+^, and Zn^2+^ ions in Alzheimer’s disease treatment. J. Inorg. Biochem..

[38] Gogna T., Housden B.E., Houldsworth A. (2024). Exploring the role of reactive oxygen species in the pathogenesis and pathophysiology of Alzheimer’s and Parkinson’s disease and the efficacy of antioxidant treatment. Antioxidants.

[39] Parameswari R.P., Ramesh G., Chidambaram S.B., Thangavelu L. (2022). Oxidative stress mediated neuroinflammation induced by chronic sleep restriction as triggers for Alzheimer’s disease. Alzheimer’s Dement..

[40] Şak B., Sousa H.B., Prior J.A. (2025). Carbon Nanomaterial-Based Electrochemical Biosensors for Alzheimer’s Disease Biomarkers: Progress, Challenges, and Future Perspectives. Biosensors.

[41] Spina E., Ferrari R.R., Pellegrini E., Colombo M., Poloni T.E., Guaita A., Davin A. (2025). Mitochondrial Alterations, Oxidative Stress, and Therapeutic Implications in Alzheimer’s Disease: A Narrative Review. Cells.

[42] Qi W., Zhu X., Wang B., Shi Y., Dong C., Shen S., Li J., Zhang K., He Y., Zhao M. (2025). Alzheimer’s Disease Digital Biomarkers Multidimensional Landscape and AI Model Scoping Review. npj Digit. Med..

[43] Zhang X., Fu M., Wang Y., Wu T. (2025). Strategies for Delivering Drugs across the Blood–Brain Barrier for the Treatment of Neurodegenerative Diseases. Front. Drug Deliv..

[44] Bor G., Hosta-Rigau L. (2023). Next Generation of Brain Cancer Nanomedicines to Overcome the Blood–Brain Barrier (BBB): Insights on Transcytosis, Perivascular Tumor Growth, and BBB Models. Adv. Ther..

[45] Li Y., Liu R., Zhao Z. (2025). Targeting brain drug delivery with macromolecules through receptor-mediated transcytosis. Pharmaceutics.

[46] Fang J., Zhang L., Wang Y., Chen M., He Y., Zhang C.Y., Zhang Y., Jiang X., Li J. (2025). Selective Peptide-Guided Transcytosis Enhances Extracellular Vesicle-Mediated siRNA Delivery Across the Blood–Brain Barrier. J. Biol. Chem..

[47] Hanumanthappa R., Parthasarathy A., Heggannavar G.B., Pc K., Nanjaiah H., Kumbhar R., Devaraju K.S. (2024). Recent advances in therapeutic strategies for Alzheimer’s and Parkinson’s disease using protein/peptide co-modified polymer nanoparticles. Neuroprotection.

[48] Xiang Y., Gu Q., Liu D. (2025). Brain Endothelial Cells in Blood—Brain Barrier Regulation and Neurological Therapy. Int. J. Mol. Sci..

[49] Bashir D.J., Bhat K.A., Bashir M. (2025). Deciphering Alzheimer’s Disease: Molecular Mechanisms, Preclinical Models and Strategies to Overcome the Blood–Brain Barrier. Brain Res..

[50] Ashirov O., Azimova S., Angelov B., Angelova A. (2025). Engineering Liposomes for Neurodegenerative Diseases: Targeted Delivery and Regenerative Potential. ChemNanoMat.

[51] Chen J.H., Zhan L.J., Lv C.M., Teng J.B., Cao C.Y. (2025). Advances and Challenges in Adeno-Associated Virus Gene Therapy Applications of Localized Delivery Strategies. Curr. Med. Sci..

[52] Awlqadr F.H., Noreen S., Altemimi A.B., Mohammed O.A., Qadir S.A., Ahmed D.H., Alkanan Z.T., Tsakali E., Van Impe J.F., Kozak D. (2025). Advancement in nanobubble technology: Enhancing drug and nutraceutical delivery with focus on bioavailability, targeted therapy, safety, and sustainability. Front. Nanotechnol..

[53] Eldufani J., Blaise G. (2019). The Role of Acetylcholinesterase Inhibitors Such as Neostigmine and Rivastigmine on Chronic Pain and Cognitive Function in Aging: A Review of Recent Clinical Applications. Alzheimer’s Dement. Transl. Res. Clin. Interv..

[54] Liu L., He H., Du B., He Y. (2025). Nanoscale drug formulations for the treatment of Alzheimer’s disease progression. RSC Adv..

[55] Egunlusi A.O., Joubert J. (2024). NMDA receptor antagonists: Emerging insights into molecular mechanisms and clinical applications in neurological disorders. Pharmaceuticals.

[56] Haddad H.W., Malone G.W., Comardelle N.J., Degueure A.E., Kaye A.M., Kaye A.D. (2022). Aducanumab, a novel anti-amyloid monoclonal antibody, for the treatment of Alzheimer’s disease: A comprehensive review. Health Psychol. Res..

[57] Birajdar S.V., Mazahir F., Alam M.I., Kumar A., Yadav A.K. (2023). Repurposing and clinical attributes of antidiabetic drugs for the treatment of neurodegenerative disorders. Eur. J. Pharmacol..

[58] Grabowska W., Bijak M., Szelenberger R., Gorniak L., Podogrocki M., Harmata P., Cichon N. (2025). Acetylcholinesterase as a multifunctional target in amyloid-driven neurodegeneration: From dual-site inhibitors to anti-aggregation strategies. Int. J. Mol. Sci..

[59] Skoczeń A., Rutecka N., Kaczmarek B., Kuśnierz-Gibała A., Kruk A., Kulesza M., Wawrzynów W., Miłoś M., Dorosz A., Jakubowska M.M. (2025). Advancements in Alzheimer’s disease therapy: The role of anti-amyloid monoclonal antibodies—A literature review. J. Educ. Health Sport.

[60] Aljuhani M., Ashraf A., Edison P. (2024). Evaluating clinical meaningfulness of anti-β-amyloid therapies amidst amyloid-related imaging abnormalities concern in Alzheimer’s disease. Brain Commun..

[61] Ashmawy R.E., Okesanya O.J., Ukoaka B.M., Daniel F.M., Ezedigwe S.G., Agboola A.O., Ahmed M.M., Ogaya J.B., Amisu B.O., Adigun O.A. (2025). Exploring the efficacy and safety of lecanemab in early Alzheimer’s disease: A systematic review of clinical evidence. J. Alzheimer’s Dis..

[62] Palaniyappan R., Arthanari S., Subramanian M., Periyasamy P., Pethappachetty P., Ganesan R., Prakash M., Govindaraj A. (2024). Nanotechnology-driven approaches in overcoming drug delivery challenges for neurodegenerative diseases. Indian J. Pharm. Educ. Res..

[63] Sharma D.K. (2025). Nanotechnology-driven approaches for early detection and targeted treatment of Alzheimer’s disease. J. Drug Target..

[64] Harwansh R.K., Deshmukh R., Barkat M.A., Rahman M.A. (2019). Bioinspired Polymeric-Based Core–Shell Smart Nano-Systems. Pharm. Nanotechnol..

[65] Guo Y., Li N., Zhang D., Gu J., Liao Z., Teng Z., Du X., Timashev P.S., Chen S., Huo S. (2025). Advancing Design Strategies in Smart Stimulus-Responsive Liposomes for Drug Release and Nanomedicine. BMEMat.

[66] Lombardo D., Kiselev M.A., Caccamo M.T. (2019). Smart Nanoparticles for Drug Delivery Application: Development of Versatile Nanocarrier Platforms in Biotechnology and Nanomedicine. J. Nanomater..

[67] Bchkol D.H.K., Mohammed O.S. (2025). Review of controlled drug delivery methods, dosage form categorization, and therapeutic impact on the human body. Span. J. Innov. Integr..

[68] Cojocaru E., Petriș O.R., Cojocaru C. (2024). Nanoparticle-Based Drug Delivery Systems in Inhaled Therapy: Improving Respiratory Medicine. Pharmaceuticals.

[69] Liu Q., Chen Z., Guiseppi-Elie A., Meng F., Luo L. (2025). Recent progress on nanotechnologies for enhancing blood–brain barrier permeability. Smart Mol..

[70] Haro-Martínez E., Muscolino E., Moral N., Duran J., Fornaguera C. (2025). Crossing the blood–brain barrier: Nanoparticle-based strategies for neurodegenerative disease therapy. Drug Deliv. Transl. Res..

[71] Zhou M., Wen K., Bi Y., Lu H., Chen J., Hu Y., Chai Z. (2017). The Application of Stimuli-Responsive Nanocarriers for Targeted Drug Delivery. Curr. Top. Med. Chem..

[72] Dehghani P., Rad M.E., Zarepour A., Sivakumar P.M., Zarrabi A. (2023). An Insight into the Polymeric Nanoparticles Applications in Diabetes Diagnosis and Treatment. Mini Rev. Med. Chem..

[73] Ciftci F., Özarslan A.C., Kantarci I.C., Yelkenci A., Tavukcuoglu O., Ghorbanpour M. (2025). Advances in drug targeting, drug delivery, and nanotechnology applications: Therapeutic significance in cancer treatment. Pharmaceutics.

[74] Kirit E., Gokce C., Altun B., Yilmazer A. (2025). Nanotherapeutic strategies for overcoming the blood–brain barrier: Applications in disease modeling and drug delivery. ACS Omega.

[75] Isik S., Osman S., Yeman-Kiyak B., Shamshir S.R.M., Sanchez N.M.E. (2025). Advances in neurodegenerative disease therapy: Stem cell clinical trials and promise of engineered exosomes. CNS Neurosci. Ther..

[76] Li C., Yao S., Li Z., Gao Y. (2025). Application of novel drug-delivery strategies in neurological disorders. Adv. Mater..

[77] Elsayed L.A., Saif A.M., Elghol S.E., Zayed M.N., Amin Y.M., Omran M.H., Ragab M.A., Althobiti R.A., Ali G.A. (2025). A comprehensive review of promising phytoconstituents as anti-cancer agents: Biological mechanisms and applications across different cancers. Curr. Nanosci..

[78] Patel M., Minglani V.V., Vaddadi H., Jha L.L., Patel L.D., Huanbutta K., Sangnim T. (2025). Advancements in nanotherapeutics for the treatment of depression via intranasal pathway: A review. Int. J. Nanomed..

[79] Hassan A.A., Ramadan E., Kristó K., Regdon G., Sovány T. (2025). Lipid–polymer hybrid nanoparticles as a smart drug delivery system for peptide/protein delivery. Pharmaceutics.

[80] Islam A., Mehwish S., Riaz F., Khan A.U. (2025). Applications and safety of biosynthesized nanoparticles in neurological disorders. Expanding Nanobiotechnology: Applications and Commercialization.

[81] Liu G., Sun Y. (2025). Harnessing nanoparticles for Alzheimer’s disease: Innovations in drug delivery and pathology-specific treatments. J. Alzheimer’s Dis..

[82] Nguyen T.T.L., Duong V.A. (2025). Advancements in nanocarrier systems for nose-to-brain drug delivery. Pharmaceuticals.

[83] Modgil M., Sharma A. (2025). Emerging era in colloidal carriers approach for enhanced transdermal drug delivery. Curr. Nanosci..

[84] Pinkiewicz M., Zaczyński A., Walecki J., Zawadzki M. (2025). Beyond the walls of Troy: A scoping review on pharmacological strategies to enhance drug delivery across the blood–brain barrier and blood–tumor barrier. Int. J. Mol. Sci..

[85] Zou Y., Zhang J., Chen L., Xu Q., Yao S., Chen H. (2025). Targeting neuroinflammation in central nervous system diseases by oral delivery of lipid nanoparticles. Pharmaceutics.

[86] Kochman U., Sitka H., Kuźniar J., Czaja M., Kozubek P., Beszłej J.A., Leszek J. (2026). Targeted Nanoparticles for Drug Delivery across the Blood–Brain Barrier in Early and Late Stages of Alzheimer’s Disease: A Review. Mol. Neurobiol..

[87] Kalra V., Silakari O., Tiwary A.K. (2025). Intranasal administration of in silico designed Rivastigmine mucoadhesive nanoparticles ameliorates scopolamine-induced Alzheimer’s symptoms in mice: Pharmacokinetic and pharmacodynamic evidence. Int. J. Pharm..

[88] Maurya R., Vikal A., Patel P., Narang R.K., Kurmi B.D. (2024). Enhancing Oral Drug Absorption: Overcoming Physiological and Pharmaceutical Barriers for Improved Bioavailability. AAPS PharmSciTech.

[89] Tayyab S., Feroz S.R. (2021). Serum Albumin: Clinical Significance of Drug Binding and Development as Drug Delivery Vehicle. Adv. Protein Chem. Struct. Biol..

[90] De Gaetano F., Totaro N., Ventura C.A. (2025). Cyclodextrin-based formulations as a promising strategy to overcome the blood–brain barrier: Historical overview and prospects in glioblastoma treatment. Pharmaceuticals.

[91] Kumar A., Shukla R. (2025). Current strategic arsenal and advances in nose-to-brain nanotheranostics for therapeutic intervention of glioblastoma multiforme. J. Biomater. Sci. Polym. Ed..

[92] Ding L., Kshirsagar P., Agrawal P., Murry D.J. (2025). Crossing the blood–brain barrier: Innovations in receptor- and transporter-mediated transcytosis strategies. Pharmaceutics.

[93] Liu S., Jin X., Ge Y., Dong J., Liu X., Pei X., Wang P., Wang B., Chang Y., Yu X.A. (2025). Advances in brain-targeted delivery strategies and natural product-mediated enhancement of blood–brain barrier permeability. J. Nanobiotechnol..

[94] Sangavi R., Khute S., Subash P. (2025). Advances in novel drug delivery systems: A focus on nanoparticles and mucoadhesive technologies. Drug Dev. Ind. Pharm..

[95] Sabzehali S. (2025). Nanomaterials in drug delivery systems: Challenges and perspectives. Eurasian J. Chem. Med. Pet. Res..

[96] Zamanian M.Y., Khachatryan L.G., Heidari M., Darabi R., Golmohammadi M., Al-Aouadi R.F.A., Akkol E.K. (2025). The therapeutic potential of flavonols in Alzheimer’s disease: Inhibiting amyloid-β, oxidative stress, and neuroinflammation. BioFactors.

[97] Mouhamed A.A., Orensa K., Mekhail M.O., Abdelaziz N.I., Mahmoud A.M., El Mously D.A. (2025). Synthesis and characterization of core–shell mussel-inspired magnetic molecularly imprinted polymer nanoparticles for the solid-phase extraction of levofloxacin in human plasma. BMC Chem..

[98] Mehandole A., Walke N., Mahajan S., Aalhate M., Maji I., Gupta U., Mehra N.K., Singh P.K. (2023). Core–shell type lipidic and polymeric nanocapsules: The transformative multifaceted delivery systems. AAPS PharmSciTech.

[99] Rajkumar M., Govindaraj P., Vimala K., Thangaraj R., Kannan S. (2024). Chitosan/PLA-Loaded Magnesium Oxide Nanocomposite to Attenuate Oxidative Stress, Neuroinflammation and Neurotoxicity in Rat Models of Alzheimer’s Disease. Metab. Brain Dis..

[100] Bramhe P., Waghmare S., Rarokar N., Sabale P., Khedekar P., Sabale V., Potey L. (2025). Polymer Blends Innovation: Advancement in Novel Drug Delivery. Int. J. Polym. Mater. Polym. Biomater..

[101] Espinoza S.M., Patil H.I., San Martin Martinez E., Casanas Pimentel R., Ige P.P. (2020). Poly-ε-caprolactone (PCL), a promising polymer for pharmaceutical and biomedical applications: Focus on nanomedicine in cancer. Int. J. Polym. Mater. Polym. Biomater..

[102] Eltaib L. (2025). Polymeric nanoparticles in targeted drug delivery: Unveiling the impact of polymer characterization and fabrication. Polymers.

[103] Phuna Z.X., Vijayabalan S., Panda B.P., Shivashekaregowda N.K.H., Shaikh M.F., Madhavan P. (2025). Improved cognition and memory via PLGA nanoparticle-mediated delivery of curcumin and piperine in an in vivo Alzheimer’s disease model. Drug Deliv. Transl. Res..

[104] Youssef Y.A., Tammam S.N., Elshenawy B.M., Ilyas S., Gad A.A., Farag K.S., Mathur S., Abdel-Kader R.M. (2025). Peptide-loaded chitosan nanoparticles improve mitochondrial and cognitive functions via inhibition of Aβ-ABAD interaction in Alzheimer’s disease. Eur. J. Pharm. Biopharm..

[105] Kömür M., Kıyan H.T., Öztürk A.A. (2025). Development of donepezil hydrochloride-loaded PLGA-based nanoparticles for Alzheimer’s disease treatment. Sci. Rep..

[106] Kouhjani M., Jaafari M.R., Saberi A., Gholami L., Tafaghodi M. (2025). Nose to brain delivery of insulin loaded in PLGA and chitosan-coated PLGA nanoparticles: A promising approach for Alzheimer’s disease therapy. J. Drug Deliv. Sci. Technol..

[107] Pawar S., Suvarna V. (2025). Curcumin-loaded PEG-functionalized carbon nanotubes: A novel strategy for Alzheimer’s management. Ther. Deliv..

[108] Qian K., Bao X., Li Y., Wang P., Guo Q., Yang P., Xu S., Yu F., Meng R., Cheng Y. (2022). Cholinergic neuron targeting nanosystem delivering hybrid peptide for combinatorial mitochondrial therapy in Alzheimer’s disease. ACS Nano.

[109] Imam F., Mukhopadhyay S., Kothiyal P., Alshehri S., Alharbi K.S., Afzal M., Iqbal M., Khan M.R., Anwer M.K., Alanazi A.A.H. (2024). Formulation and characterization of polymeric nanoparticle of Rivastigmine for effective management of Alzheimer’s disease. Saudi Pharm. J..

[110] Mahanta A.K., Chaulagain B., Trivedi R., Singh J. (2024). Mannose-functionalized chitosan-coated PLGA nanoparticles for brain-targeted codelivery of CBD and BDNF for the treatment of Alzheimer’s disease. ACS Chem. Neurosci..

[111] Mohammadbaghban E., Taravati A., Najafzadehvarzi H., Khaleghzadeh-Ahangar H., Tohidi F. (2024). Oral administration of encapsulated catechin in chitosan–alginate nanoparticles improves cognitive function and neurodegeneration in an aluminum chloride-induced rat model of Alzheimer’s disease. Physiol. Rep..

[112] Zameer S., Ali J., Vohora D., Najmi A.K., Akhtar M. (2021). Development, Optimisation and Evaluation of Chitosan Nanoparticles of Alendronate against Alzheimer’s Disease in Intracerebroventricular Streptozotocin Model for Brain Delivery. J. Drug Target..

[113] Shahidi S., Asl S.S., Gholamigeravand B., Afshar S., Hashemi-Firouzi N., Samzadeh-Kermani A., Majidi M., Amiri K. (2024). Effect of Mesenchymal Stem Cells and Polyvinyl Alcohol-Coated Selenium Nanoparticles on Rats with Alzheimer-Like Phenotypes. Iran. J. Basic Med. Sci..

[114] Hashemi-Firouzi N., Afshar S., Asl S.S., Samzadeh-Kermani A., Gholamigeravand B., Amiri K., Majidi M., Shahidi S. (2022). The Effects of Polyvinyl Alcohol-Coated Selenium Nanoparticles on Memory Impairment in Rats. Metab. Brain Dis..

[115] Oliveira Silva R., Counil H., Rabanel J.-M., Haddad M., Zaouter C., Ben Khedher M.R., Patten S.A., Ramassamy C. (2024). Donepezil-loaded nanocarriers for the treatment of Alzheimer’s disease: Superior efficacy of extracellular vesicles over polymeric nanoparticles. Int. J. Nanomed..

[116] Ediriweera G.R., Chen L., Yerbury J.J., Thurecht K.J., Vine K.L. (2021). Non-viral vector-mediated gene therapy for ALS: Challenges and future perspectives. Mol. Pharm..

[117] Kushawaha S.K., Ashawat M.S., Arora R., Baldi A. (2025). Auranofin-loaded PLGA nanoparticles for neuroprotection against aluminium-induced Alzheimer’s disease. Curr. Pharm. Des..

[118] Handa M., Sanap S.N., Bhatta R.S., Patil G.P., Ghose S., Singh D.P., Shukla R. (2023). Combining donepezil and memantine via mannosylated PLGA nanoparticles for intranasal delivery: Characterization and preclinical studies. Biomater. Adv..

[119] Puri A., Mohite P., Munde S., Ade N., Ramole A., Pillai D., Nagare S., Mahadik S., Singh S. (2025). Unlocking the Multifaceted Potential of Lipid-Based Dispersion as a Drug Carrier: Targeted Applications and Stability Improvement Strategies. J. Dispers. Sci. Technol..

[120] Kim W., Han J., Chauhan S., Lee J.W. (2025). Cell-Free Protein Synthesis and Vesicle Systems for Programmable Therapeutic Manufacturing and Delivery. J. Biol. Eng..

[121] Song Q., Li J., Li T., Li H.W. (2024). Nanomaterials That Aid in the Diagnosis and Treatment of Alzheimer’s Disease, Resolving Blood–Brain Barrier Crossing Ability. Adv. Sci..

[122] Senapati S., Tripathi K., Awad K., Rahimipour S. (2024). Multifunctional liposomes targeting amyloid-β oligomers for early diagnosis and therapy of Alzheimer’s disease. Small.

[123] He X., Peng Y., Huang S., Xiao Z., Li G., Zuo Z., Zhang L., Shuai X., Zheng H., Hu X. (2024). Blood–brain barrier-crossing delivery of felodipine nanodrug ameliorates anxiety-like behavior and cognitive impairment in Alzheimer’s disease. Adv. Sci..

[124] Xu Y., Ye X., Du Y., Yang W., Tong F., Li W., Huang Q., Chen Y., Li H., Gao H. (2025). Nose-to-brain delivery of targeted lipid nanoparticles as two-pronged β-amyloid nanoscavenger for Alzheimer’s disease therapy. Acta Pharm. Sin. B.

[125] Attia R.T., Fahmy S.A., Abdel-Latif R.T., Ateyya H., Fayed T.W., El-Abhar H.S. (2025). Cationic lipid-based nanoparticles-formulated artesunate as a neurotherapeutic agent in Alzheimer’s disease: Targeting inflammasome activation and pyroptosis pathway. J. Drug Deliv. Sci. Technol..

[126] Abla K.K., Torossian T., Ammar J., Karam M., Fawaz M., Debian R., Mehanna M., Daou A., Mhanna R. (2025). Double-layered nanovesicular chitosan-coated liposomal system loaded with donepezil cargo for Alzheimer’s disease: Design, optimization, and in vivo evaluation. Int. J. Biol. Macromol..

[127] Zhang H., Jiang W., Zhao Y., Song T., Xi Y., Han G., Jin Y., Song M., Bai K., Zhou J. (2022). Lipoprotein-inspired nanoscavenger for the three-pronged modulation of microglia-derived neuroinflammation in Alzheimer’s disease therapy. Nano Lett..

[128] Lin R.R., Jin L.L., Xue Y.Y., Zhang Z.S., Huang H.F., Chen D.F., Liu Q., Mao Z.W., Wu Z.Y., Tao Q.Q. (2024). Hybrid membrane-coated nanoparticles for precise targeting and synergistic therapy in Alzheimer’s disease. Adv. Sci..

[129] Far B.F., Safaei M., Pourmolaei A., Adibamini S., Shirdel S., Shirdel S., Emadi R., Kaushik A.K. (2024). Exploring curcumin-loaded lipid-based nanomedicine as efficient targeted therapy for Alzheimer’s disease. ACS Appl. Bio Mater..

[130] Shan Q., Zhi Y., Chen Y., Yao W., Zhou H., Che J., Bai F. (2024). Intranasal liposomes co-delivery of Aβ-targeted KLVFF and ROS-responsive ceria for synergistic therapy of Alzheimer’s disease. Chem. Eng. J..

[131] Zhang Q., Zhang X., Yang B., Li Y., Sun X.H., Li X., Sui P., Wang Y.B., Tian S.Y., Wang C.Y. (2024). Ligustilide-loaded liposome ameliorates mitochondrial impairments and improves cognitive function via the PKA/AKAP1 signaling pathway in a mouse model of Alzheimer’s disease. CNS Neurosci. Ther..

[132] Su D., Chen Z., An X., Yang J., Yang J., Wang X., Qu Y., Gong C., Chai Y., Liu X. (2024). MicroRNA-195 liposomes for therapy of Alzheimer’s disease. J. Control. Release.

[133] Meng R., Yang X., Li Y., Zhang Q. (2024). Extending Dual-Targeting Upper-Limit in Liposomal Delivery of Lithospermic Acid B for Alzheimer’s Mitochondrial Revitalization. J. Control. Release.

[134] Andrade S., Pereira M.C., Loureiro J.A. (2023). Caffeic acid loaded into engineered lipid nanoparticles for Alzheimer’s disease therapy. Colloids Surf. B Biointerfaces.

[135] Ullah A., Khan M., Zhang Y., Shafiq M., Ullah M., Abbas A., Xianxiang X., Chen G., Diao Y. (2025). Advancing therapeutic strategies with polymeric drug conjugates for nucleic acid delivery and treatment. Int. J. Nanomed..

[136] Gul R., Jan H., Lalay G., Andleeb A., Usman H., Zainab R., Qamar Z., Hano C., Abbasi B.H. (2021). Medicinal plants and biogenic metal oxide nanoparticles: A paradigm shift to treat Alzheimer’s disease. Coatings.

[137] Bagherpour I., Mozafari M.R., Naghib S.M. (2025). Inorganic nanoparticles-based drug delivery systems for neurodegenerative diseases therapy. Curr. Pharm. Des..

[138] Mumtaz, Unnithan D., Bano A., Chauhan A.P.S., Ali J., Khan M.A. (2025). Targeting Alzheimer’s disease pathology: Influence of nano-based drug delivery systems loaded with a combination of herbal and synthetic drugs. Expert Opin. Drug Deliv..

[139] Zhang L., Cao K., Xie J., Liang X., Gong H., Luo Q., Luo H. (2024). Aβ42 and ROS dual-targeted multifunctional nanocomposite for combination therapy of Alzheimer’s disease. J. Nanobiotechnol..

[140] Chang Y.J., Chien Y.H., Chang C.C., Wang P.N., Chen Y.R., Chang Y.C. (2024). Detection of femtomolar amyloid-β peptides for early-stage identification of Alzheimer’s amyloid-β aggregation with functionalized gold nanoparticles. ACS Appl. Mater. Interfaces.

[141] Xu L., Guo M., Hung C.T., Shi X.L., Yuan Y., Zhang X., Jin R.H., Li W., Dong Q., Zhao D. (2023). Chiral skeletons of mesoporous silica nanospheres to mitigate Alzheimer’s β-amyloid aggregation. J. Am. Chem. Soc..

[142] Yin X., Zhou H., Cao T., Yang X., Meng F., Dai X., Wang Y., Li S., Zhai W., Yang Z. (2024). Rational design of dual-functionalized Gd@C82 nanoparticles to relieve neuronal cytotoxicity in Alzheimer’s disease via inhibition of Aβ aggregation. ACS Nano.

[143] Wang Y., Wang X., Xie R., Burger J.C., Tong Y., Gong S. (2023). Overcoming the Blood–Brain Barrier for Gene Therapy via Systemic Administration of GSH-Responsive Silica Nanocapsules. Adv. Mater..

[144] Noor N.A., Hosny E.N., Khadrawy Y.A., Mourad I.M., Othman A.I., Aboul Ezz H.S., Mohammed H.S. (2022). Effect of Curcumin Nanoparticles on Streptozotocin-Induced Male Wistar Rat Model of Alzheimer’s Disease. Metab. Brain Dis..

[145] Liu L., Liu W., Sun Y., Dong X. (2024). Serum albumin-embedding copper nanoclusters inhibit Alzheimer’s β-amyloid fibrillogenesis and neuroinflammation. J. Colloid Interface Sci..

[146] Yin X., Zhou H., Zhang M., Su J., Wang X., Li S., Yang Z., Kang Z., Zhou R. (2023). C3N Nanodots Inhibit Aβ Peptides Aggregation Pathogenic Path in Alzheimer’s Disease. Nat. Commun..

[147] Yin Z., Zhang Z., Gao D., Luo G., Ma T., Wang Y., Lu L., Gao X. (2022). Stepwise Coordination-Driven Metal–Phenolic Nanoparticle as a Neuroprotection Enhancer for Alzheimer’s Disease Therapy. ACS Appl. Mater. Interfaces.

[148] Yang L., Cui Y., Liang H., Li Z., Wang N., Wang Y., Zheng G. (2022). Multifunctional selenium nanoparticles with different surface modifications ameliorate neuroinflammation via the gut microbiota–NLRP3 inflammasome–brain axis in APP/PS1 mice. ACS Appl. Mater. Interfaces.

[149] Ruan Y., Xiong Y., Fang W., Yu Q., Mai Y., Cao Z., Wang K., Lei M., Xu J., Liu Y. (2022). Highly sensitive curcumin-conjugated nanotheranostic platform for detecting amyloid-beta plaques by MRI and reversing cognitive deficits via NLRP3 inhibition. J. Nanobiotechnol..

[150] Li Q.Y., Yu X., Li X., Bao L.N., Zhang Y., Xie M.J., Jiang M., Wang Y.Q., Huang K., Xu L. (2023). Silicon–carbon dots-loaded mesoporous silica nanocomposites (mSiO_2_@SiCDs): An efficient dual inhibitor of Cu^2^⁺-mediated oxidative stress and Aβ aggregation for Alzheimer’s disease. ACS Appl. Mater. Interfaces.

[151] Ge K., Mu Y., Liu M., Bai Z., Liu Z., Geng D., Gao F. (2022). Gold nanorods with spatial separation of CeO_2_ deposition for plasmonic-enhanced antioxidant stress and photothermal therapy of Alzheimer’s disease. ACS Appl. Mater. Interfaces.

[152] Zhang X., Li Y., Hu Y. (2020). Green Synthesis of Silver Nanoparticles and Their Preventive Effect in Deficits in Recognition and Spatial Memory in Sporadic Alzheimer’s Rat Model. Colloids Surf. A Physicochem. Eng. Asp..

[153] Zhang M., Chen H., Zhang W., Liu Y., Ding L., Gong J., Ma R., Zheng S., Zhang Y. (2023). Biomimetic Remodeling of Microglial Riboflavin Metabolism Ameliorates Cognitive Impairment by Modulating Neuroinflammation. Adv. Sci..

[154] Li C., Wang N., Zheng G., Yang L. (2021). Oral administration of resveratrol–selenium–peptide nanocomposites alleviates Alzheimer’s disease-like pathogenesis by inhibiting Aβ aggregation and regulating gut microbiota. ACS Appl. Mater. Interfaces.

[155] Boyuklieva R., Zahariev N., Simeonov P., Penkov D., Katsarov P. (2025). Next-generation drug delivery for neurotherapeutics: The promise of stimuli-triggered nanocarriers. Biomedicines.

[156] Hermann D.M., Bacigaluppi M., Bassetti C.L., Bassotti G., Boltze J., Chan A., Dalkara T., Denes A., Diez-Tejedor E., Dodel R. (2025). Most Prominent Challenges in Translational Neuroscience and Strategic Solutions to Bridge the Gaps: Perspectives from an Editorial Board Interrogation. Explor. Neurosci..

[157] Lu J., Dou D., Wang Y., Shu J., Lei Z., Huo Y., Gong X. (2025). Enhanced Pre- and Postnatal Developmental Toxicity (ePPND) Study in Non-Human Primates: Necessity, Strategic Approaches, and Critical Considerations. Regul. Toxicol. Pharmacol..

[158] Mangrulkar S.V., Dabhekar S.V., Neje P., Parkarwar N., Turankar A., Taksande B.G., Umekar M.J., Nakhate K.T. (2024). In Vivo Animal Models Development and Their Limitations for Brain Research. Application of Nanocarriers in Brain Delivery of Therapeutics.

[159] Gao Y., Liu Y., Li Y. (2024). Safety and Efficacy of Acetylcholinesterase Inhibitors for Alzheimer’s Disease: A Systematic Review and Meta-Analysis. Adv. Clin. Exp. Med..

[160] Chavan A.K., Tatiya A.U. (2025). Development of Novel Carrier System: A Key Approach to Enhance Bioavailability of Herbal Medicines. Pharmacogn. Res..

[161] Wang K., Yang R., Li J., Wang H., Wan L., He J. (2025). Nanocarrier-Based Targeted Drug Delivery for Alzheimer’s Disease: Addressing Neuroinflammation and Enhancing Clinical Translation. Front. Pharmacol..

[162] Georgieva D., Bogdanova I., Mihaylova R., Alexandrova M., Bozhilova S., Christova D., Kostova B. (2025). Orodispersible Hydrogel Film Technology for Optimized Galantamine Delivery in the Treatment of Alzheimer’s Disease. Gels.

[163] Tomar S., Yadav R.K., Shah K., Dewangan H.K. (2024). A Comprehensive Review on Carrier-Mediated Nose-to-Brain Targeting: Emphasis on Molecular Targets, Current Trends, Future Prospects, and Challenges. Int. J. Polym. Mater. Polym. Biomater..

[164] Graham U.M., Pinto J.M., Weuve J., Dozier A.K., Rogers R., Nag M.D., Schneider J., Kaufman J.D., Bennett D.A., Oberdörster G. (2025). Nose to Brain Translocation of Inhaled Ultrafine Elongated Particles: Facts and Mysteries. Front. Toxicol..

[165] Kuzuya M. (2023). Effect of Drugs on Nutritional Status and Drug–Nutrition Interactions in Older Patients. Geriatr. Gerontol. Int..

[166] Grover P., Thakur K., Bhardwaj M., Mehta L., Raina S.N., Rajpal V.R. (2024). Phytotherapeutics in Cancer: From Potential Drug Candidates to Clinical Translation. Curr. Top. Med. Chem..

[167] Kumarasamy R.V., Natarajan P.M., Umapathy V.R., Roy J.R., Mironescu M., Palanisamy C.P. (2024). Clinical Applications and Therapeutic Potentials of Advanced Nanoparticles: A Comprehensive Review on Completed Human Clinical Trials. Front. Nanotechnol..

